# Supplementary material for: From anxiety to action—Experience of threat, emotional states, reactance, and action preferences in the early days of COVID-19 self-isolation in Germany and Austria
Source: PLoS One. 2020 Dec 8;15(12):e0243193. doi: 10.1371/journal.pone.0243193 (PMC7723254; doi:10.1371/journal.pone.0243193)
Supplement: S1 File — (ZIP) [file pone.0243193.s001.zip › S1-Analysis-Script-incl-SEM.html]

Revision Analysis and Markdown


# Revision Analysis and Markdown

#### Stefan Reiss, Vittoria Franchina, Chiara Jutzi, Robin Willardt & Eva Jonas

#### Nov 2020

```
df<-read_csv("S1_Data.csv")%>%
distinct()%>%
mutate(education = fct_relevel(education, "bn10", after=Inf),
education = fct_relevel(education, "-oth-", after=Inf))


df<-df%>%
mutate(othaction=str_replace_all(othaction,"\\\n",""))
```

# Descriptives

## Age

```
df<-df %>%
mutate(sex = case_when(
sex == "G1" ~ "male",
sex == "G2" ~ "female",
sex == "G3" ~ "divers",
sex == "G4" ~ "undisclosed"
))
```

```
df<-df %>%
filter(country %in% c("DE", "AT")) %>%
filter(sex %in% c("male", "female"))
```

```
df%>%
ggplot(aes(x=age,fill=sex))+
geom_histogram(alpha=.5,bins=10)
```

```
psych::describe(df$age)
```

```
   vars   n mean    sd median trimmed   mad min max range skew kurtosis   se
X1    1 395 34.4 14.82     30   32.79 13.34  14  78    64 0.77    -0.61 0.75
```

```
df%>%
count(sex)
```

```
# A tibble: 2 x 2
  sex        n
  <chr>  <int>
1 female   267
2 male     128
```

##Country

```
df%>%
count(country)%>%
kable()
```

| country | n |
| --- | --- |
| AT | 126 |
| DE | 269 |

```
df<-df%>%
mutate(duration=datestamp-startdate)


df%>%ggplot(aes(x=duration))+
geom_density()+
scale_y_continuous(labels=scales::percent)
```

```
df%>%
summarise(mean=mean(duration),
med=median(duration),
min=min(duration),
max=max(duration))
```

```
# A tibble: 1 x 4
  mean          med           min       max          
  <drtn>        <drtn>        <drtn>    <drtn>       
1 22.77903 mins 16.93333 mins 7.65 mins 257.7167 mins
```

```
df%>%
ggplot(aes(x=education))+
geom_bar()
```

```
df%>%
filter(education=="-oth-")%>%
select(starts_with("edu"))
```

```
# A tibble: 7 x 2
  education `education[other]`         
  <fct>     <chr>                      
1 -oth-     Lehrerin                   
2 -oth-     Mag.                       
3 -oth-     <NA>                       
4 -oth-     Facharzt                   
5 -oth-     Staatsexamen               
6 -oth-     Examensstudium/ Universität
7 -oth-     Es lebe die nivealosigkeit
```

```
df<-df%>%
mutate(education=case_when(
education=="-oth-"~"bn1",
`education[other]`=="Lehrerin"~"bn9",
`education[other]`=="Mag."~"bn9",
`education[other]`=="Facharzt"~"bn9",
`education[other]`=="Staatsexamen"~"bn9",
`education[other]`=="Examensstudium/Universität"~"bn9",
`education[other]`=="Eslebedienivealosigkeit"~"bn1",
TRUE~as.character(education)))
```

##Risk Group

```
df%>%
count(risk,precond1)
```

```
# A tibble: 4 x 3
   risk precond1     n
  <dbl>    <dbl> <int>
1     0        0   327
2     0        1    17
3     1        0    20
4     1        1    31
```

###Educationlevels

```
df%>%
count(education)
```

```
# A tibble: 10 x 2
   education     n
   <chr>     <int>
 1 bn1           7
 2 bn10         11
 3 bn2           3
 4 bn3          10
 5 bn4          24
 6 bn5          24
 7 bn6         125
 8 bn7          28
 9 bn8          50
10 bn9         113
```

MostoftheparticipantswerebetweenHighSchoolDiplomaandMaster’sdegree

```
df<-df%>%
rename_at(vars(starts_with("discr[")),
funs(str_remove(.,"discr\\[")))%>%
rename_at(vars(starts_with("Affekt")),
funs(str_remove(.,"Affekt\\[")))%>%
rename_at(vars(`aut1]`:`basl3]`),
funs(str_remove(.,"\\]")))
```

##ActionStrategies

```
strategies<- df%>%
select(id,`sinnv[sens1]`:`coping[coping13]`)%>%
pivot_longer(cols=`sinnv[sens1]`:`coping[coping13]`,names_to="strategy",values_to="value")%>%
mutate(strat_type=parse_number(strategy),
judge=str_extract(strategy,pattern="\\[(....)"),
judge=str_remove(judge,"\\["))%>%
mutate(action=case_when(
strat_type==1~"Stockpiling",
strat_type==2~"WashingHands",
strat_type==3~"Spiritual",
strat_type==4~"Creative",
strat_type==5~"OwnProjects",
strat_type==6~"EntertainNeighbors",
strat_type==7~"DigitalSocialContacts",
strat_type==8~"Information",
strat_type==9~"SocialMedia",
strat_type==10~"GluedtoTV",
strat_type==11~"HouseholdQualityTime",
strat_type==12~"DisregardCurfew",
strat_type==13~"DoingSports"
))


strat_df<-strategies %>% left_join(df %>% select(id, age))
```

```
strat_rate<-strat_df%>%
pivot_wider(id_cols=c("id","action"),names_from="judge",values_from="value")

(strat<-psych::alpha(strat_rate %>% select(sens:copi)))
```

```
Reliability analysis   
Call: psych::alpha(x = strat_rate %>% select(sens:copi))

  raw_alpha std.alpha G6(smc) average_r S/N    ase mean  sd median_r
      0.91      0.91    0.87      0.77 9.8 0.0022  2.8 1.3     0.76

 lower alpha upper     95% confidence boundaries
0.9 0.91 0.91 

 Reliability if an item is dropped:
     raw_alpha std.alpha G6(smc) average_r S/N alpha se var.r med.r
sens      0.86      0.86    0.75      0.75 6.1   0.0039    NA  0.75
inte      0.88      0.88    0.79      0.79 7.5   0.0033    NA  0.79
copi      0.86      0.86    0.76      0.76 6.2   0.0039    NA  0.76

 Item statistics 
        n raw.r std.r r.cor r.drop mean  sd
sens 5135  0.92  0.92  0.87   0.82  2.9 1.5
inte 5135  0.91  0.91  0.84   0.80  2.8 1.4
copi 5135  0.92  0.92  0.87   0.82  2.7 1.4

Non missing response frequency for each item
        1    2    3    4    5 miss
sens 0.26 0.16 0.17 0.23 0.17    0
inte 0.26 0.18 0.18 0.22 0.16    0
copi 0.30 0.18 0.18 0.21 0.13    0
```

```
strat_rate$strategies <- strat$scores


strat_df<-strat_df%>%
left_join(strat_rate)
```

```
df<-strat_rate%>%
pivot_wider(id_cols=c("id"),names_from="action",values_from="strategies")%>%
janitor::clean_names()%>%
right_join(df)
```

```
df<-df%>%
select(SJ=starts_with('System[SJ'))%>%
cbind(df)
```

```
#System Justification

(sysj<-alpha(df%>%select(contains("System")),check.keys=T))
```

```
Reliability analysis   
Call: alpha(x = df %>% select(contains("System")), check.keys = T)

  raw_alpha std.alpha G6(smc) average_r S/N   ase mean   sd median_r
      0.75      0.75    0.76      0.28 3.1 0.019  4.7 0.95     0.28

 lower alpha upper     95% confidence boundaries
0.71 0.75 0.79 

 Reliability if an item is dropped:
             raw_alpha std.alpha G6(smc) average_r S/N alpha se var.r med.r
System[SJ1]       0.69      0.69    0.69      0.25 2.3    0.024 0.011  0.24
System[SJ2]       0.71      0.71    0.71      0.26 2.5    0.022 0.014  0.24
System[SJ3]-      0.74      0.74    0.74      0.29 2.8    0.020 0.012  0.30
System[SJ4]       0.73      0.73    0.74      0.28 2.7    0.021 0.017  0.30
System[SJ5]       0.74      0.74    0.75      0.29 2.9    0.020 0.013  0.29
System[SJ6]       0.73      0.73    0.73      0.28 2.7    0.020 0.015  0.28
System[SJ7]-      0.73      0.73    0.73      0.28 2.7    0.021 0.013  0.29
System[SJ8]       0.73      0.74    0.74      0.29 2.8    0.020 0.015  0.30

 Item statistics 
               n raw.r std.r r.cor r.drop mean  sd
System[SJ1]  395  0.76  0.74  0.73   0.64  4.0 1.6
System[SJ2]  395  0.65  0.67  0.61   0.51  5.1 1.5
System[SJ3]- 395  0.59  0.56  0.47   0.40  4.7 1.8
System[SJ4]  395  0.59  0.60  0.51   0.44  5.3 1.5
System[SJ5]  395  0.50  0.54  0.43   0.36  6.0 1.2
System[SJ6]  395  0.59  0.59  0.50   0.42  4.7 1.7
System[SJ7]- 395  0.60  0.59  0.52   0.44  4.7 1.6
System[SJ8]  395  0.57  0.56  0.46   0.40  3.2 1.6

Non missing response frequency for each item
               1    2    3    4    5    6    7 miss
System[SJ1] 0.06 0.11 0.21 0.20 0.24 0.11 0.07    0
System[SJ2] 0.03 0.03 0.10 0.12 0.27 0.27 0.17    0
System[SJ3] 0.20 0.20 0.16 0.13 0.16 0.07 0.06    0
System[SJ4] 0.02 0.02 0.07 0.15 0.22 0.26 0.26    0
System[SJ5] 0.01 0.01 0.04 0.05 0.13 0.32 0.43    0
System[SJ6] 0.06 0.06 0.12 0.16 0.23 0.22 0.16    0
System[SJ7] 0.14 0.22 0.19 0.23 0.14 0.05 0.04    0
System[SJ8] 0.16 0.22 0.21 0.18 0.15 0.05 0.02    0
```

```
df$sysjust<-sysj$scores
```

Scale all variables

```
dfs<-df%>%
mutate_if(is.numeric,scale,center=T,scale=T)
```

#Structural Equation Model

```
pacman::p_load(tidyverse,knitr,lavaan,tictoc)
```

```
package 'tictoc' successfully unpacked and MD5 sums checked

The downloaded binary packages are in
    C:\Users\b1003556\AppData\Local\Temp\Rtmp2bJiCQ\downloaded_packages
```

##Prepare and rename data

```
df<-df%>%
rename_at(vars(starts_with("handlint[")),
funs(str_remove(.,"handlint\\[")))%>%
rename_at(vars(starts_with("sinnv[")),
funs(str_remove(.,"sinnv\\[")))%>%
rename_at(vars(starts_with("coping[")),
funs(str_remove(.,"coping\\[")))%>%
rename_at(vars(`sens1]`:`coping13]`),
funs(str_remove(.,"\\]")))

dfs<-dfs%>%
rename_at(vars(starts_with("handlint[")),
funs(str_remove(.,"handlint\\[")))%>%
rename_at(vars(starts_with("sinnv[")),
funs(str_remove(.,"sinnv\\[")))%>%
rename_at(vars(starts_with("coping[")),
funs(str_remove(.,"coping\\[")))%>%
rename_at(vars(`sens1]`:`coping13]`),
funs(str_remove(.,"\\]")))
```

```
df<-df%>%
rename_at(vars(starts_with("lonel[")),
funs(str_remove(.,"lonel\\[")))%>%
rename_at(vars(`lsoc1]`:`lrom5]`),
funs(str_remove(.,"\\]")))
```

#Measurement models to analyze scale consistency

## Discrepancy Scale

```
disc.mod<-"
discr=~aut2+aut1+aut3+aut4+
uncer1+uncer2+uncer3+uncer4+
agenc1+agenc2+agenc3+agenc4

exp=~expec1+expec2+expec3


#Covariances
aut1~~aut2
aut1~~aut3
aut1~~aut4
aut2~~aut3
aut2~~aut4
aut3~~aut4

uncer1~~uncer2
uncer1~~uncer3
uncer1~~uncer4
uncer2~~uncer3
uncer2~~uncer4
uncer3~~uncer4

agenc1~~agenc2
agenc1~~agenc3
agenc1~~agenc4
agenc2~~agenc3
agenc2~~agenc4
agenc3~~agenc4

"


#+expec1+expec2+expec3
```

```
disc.cfa<-cfa(disc.mod,data=dfs)

disc.sum<-summary(disc.cfa,fit.measures=TRUE,
standardized=TRUE)
```

```
lavaan 0.6-7 ended normally after 46 iterations

  Estimator                                         ML
  Optimization method                           NLMINB
  Number of free parameters                         49
                                                      
  Number of observations                           395
                                                      
Model Test User Model:
                                                      
  Test statistic                               185.523
  Degrees of freedom                                71
  P-value (Chi-square)                           0.000

Model Test Baseline Model:

  Test statistic                              1697.891
  Degrees of freedom                               105
  P-value                                        0.000

User Model versus Baseline Model:

  Comparative Fit Index (CFI)                    0.928
  Tucker-Lewis Index (TLI)                       0.894

Loglikelihood and Information Criteria:

  Loglikelihood user model (H0)              -7643.517
  Loglikelihood unrestricted model (H1)      -7550.756
                                                      
  Akaike (AIC)                               15385.035
  Bayesian (BIC)                             15580.000
  Sample-size adjusted Bayesian (BIC)        15424.523

Root Mean Square Error of Approximation:

  RMSEA                                          0.064
  90 Percent confidence interval - lower         0.053
  90 Percent confidence interval - upper         0.075
  P-value RMSEA <= 0.05                          0.021

Standardized Root Mean Square Residual:

  SRMR                                           0.053

Parameter Estimates:

  Standard errors                             Standard
  Information                                 Expected
  Information saturated (h1) model          Structured

Latent Variables:
                   Estimate  Std.Err  z-value  P(>|z|)   Std.lv  Std.all
  discr =~                                                              
    aut2              1.000                               0.446    0.447
    aut1             -1.140    0.127   -8.946    0.000   -0.509   -0.509
    aut3              1.126    0.149    7.571    0.000    0.503    0.503
    aut4              0.883    0.115    7.681    0.000    0.394    0.394
    uncer1           -1.324    0.198   -6.699    0.000   -0.591   -0.591
    uncer2            0.930    0.171    5.432    0.000    0.415    0.416
    uncer3            0.833    0.166    5.029    0.000    0.372    0.372
    uncer4            1.838    0.238    7.708    0.000    0.820    0.821
    agenc1            1.193    0.168    7.107    0.000    0.533    0.533
    agenc2            0.974    0.154    6.309    0.000    0.435    0.435
    agenc3           -1.303    0.175   -7.436    0.000   -0.582   -0.582
    agenc4           -1.240    0.171   -7.251    0.000   -0.553   -0.554
  exp =~                                                                
    expec1            1.000                               0.730    0.731
    expec2           -1.025    0.089  -11.555    0.000   -0.749   -0.750
    expec3           -1.021    0.088  -11.551    0.000   -0.745   -0.746

Covariances:
                   Estimate  Std.Err  z-value  P(>|z|)   Std.lv  Std.all
 .aut2 ~~                                                               
   .aut1             -0.304    0.045   -6.699    0.000   -0.304   -0.396
 .aut1 ~~                                                               
   .aut3             -0.091    0.042   -2.181    0.029   -0.091   -0.123
   .aut4             -0.316    0.046   -6.870    0.000   -0.316   -0.401
 .aut2 ~~                                                               
   .aut3              0.125    0.043    2.921    0.003    0.125    0.162
   .aut4              0.330    0.047    6.997    0.000    0.330    0.403
 .aut3 ~~                                                               
   .aut4              0.065    0.043    1.519    0.129    0.065    0.082
 .uncer1 ~~                                                             
   .uncer2            0.087    0.046    1.900    0.057    0.087    0.119
   .uncer3            0.032    0.046    0.685    0.493    0.032    0.042
   .uncer4            0.116    0.045    2.592    0.010    0.116    0.253
 .uncer2 ~~                                                             
   .uncer3            0.033    0.048    0.701    0.483    0.033    0.040
   .uncer4           -0.082    0.044   -1.882    0.060   -0.082   -0.158
 .uncer3 ~~                                                             
   .uncer4           -0.045    0.043   -1.032    0.302   -0.045   -0.085
 .agenc1 ~~                                                             
   .agenc2            0.029    0.042    0.678    0.498    0.029    0.038
   .agenc3           -0.120    0.042   -2.854    0.004   -0.120   -0.174
   .agenc4           -0.125    0.042   -2.957    0.003   -0.125   -0.178
 .agenc2 ~~                                                             
   .agenc3           -0.070    0.042   -1.666    0.096   -0.070   -0.096
   .agenc4           -0.116    0.043   -2.720    0.007   -0.116   -0.156
 .agenc3 ~~                                                             
   .agenc4            0.182    0.043    4.270    0.000    0.182    0.269
  discr ~~                                                              
    exp               0.024    0.020    1.207    0.227    0.075    0.075

Variances:
                   Estimate  Std.Err  z-value  P(>|z|)   Std.lv  Std.all
   .aut2              0.798    0.060   13.308    0.000    0.798    0.800
   .aut1              0.739    0.057   12.956    0.000    0.739    0.740
   .aut3              0.745    0.057   12.998    0.000    0.745    0.747
   .aut4              0.842    0.062   13.522    0.000    0.842    0.844
   .uncer1            0.649    0.062   10.455    0.000    0.649    0.650
   .uncer2            0.825    0.066   12.489    0.000    0.825    0.827
   .uncer3            0.859    0.067   12.829    0.000    0.859    0.861
   .uncer4            0.325    0.058    5.611    0.000    0.325    0.326
   .agenc1            0.714    0.057   12.596    0.000    0.714    0.716
   .agenc2            0.809    0.061   13.269    0.000    0.809    0.811
   .agenc3            0.659    0.055   12.093    0.000    0.659    0.661
   .agenc4            0.691    0.056   12.401    0.000    0.691    0.693
   .expec1            0.464    0.050    9.251    0.000    0.464    0.466
   .expec2            0.437    0.050    8.662    0.000    0.437    0.438
   .expec3            0.442    0.050    8.766    0.000    0.442    0.443
    discr             0.199    0.047    4.262    0.000    1.000    1.000
    exp               0.533    0.073    7.272    0.000    1.000    1.000
```

```
disc.sum$PE%>%
filter(op=="=~")%>%
mutate(Parameter=paste(lhs,op,rhs))%>%
select(Parameter,b=est,SE=se,z,beta=std.all,p=pvalue)%>%
kable(digits=3)#%>%
```

| Parameter | b | SE | z | beta | p |
| --- | --- | --- | --- | --- | --- |
| discr =~ aut2 | 1.000 | 0.000 | NA | 0.447 | NA |
| discr =~ aut1 | -1.140 | 0.127 | -8.946 | -0.509 | 0 |
| discr =~ aut3 | 1.126 | 0.149 | 7.571 | 0.503 | 0 |
| discr =~ aut4 | 0.883 | 0.115 | 7.681 | 0.394 | 0 |
| discr =~ uncer1 | -1.324 | 0.198 | -6.699 | -0.591 | 0 |
| discr =~ uncer2 | 0.930 | 0.171 | 5.432 | 0.416 | 0 |
| discr =~ uncer3 | 0.833 | 0.166 | 5.029 | 0.372 | 0 |
| discr =~ uncer4 | 1.838 | 0.238 | 7.708 | 0.821 | 0 |
| discr =~ agenc1 | 1.193 | 0.168 | 7.107 | 0.533 | 0 |
| discr =~ agenc2 | 0.974 | 0.154 | 6.309 | 0.435 | 0 |
| discr =~ agenc3 | -1.303 | 0.175 | -7.436 | -0.582 | 0 |
| discr =~ agenc4 | -1.240 | 0.171 | -7.251 | -0.554 | 0 |
| exp =~ expec1 | 1.000 | 0.000 | NA | 0.731 | NA |
| exp =~ expec2 | -1.025 | 0.089 | -11.555 | -0.750 | 0 |
| exp =~ expec3 | -1.021 | 0.088 | -11.551 | -0.746 | 0 |

```
#kableExtra::save_kable(file="table1.png")
```

##AffectScalesCFA

```
aff.mod<-"
BIS=~fear2+fear5+fear6+bis1+bis2+bis3+bis4+bis5
react=~reac2+reac1+reac3+reac4+reac5+reac6+reac7+host1+host2+host3+host4+host5+host6
BAS=~bash1+bash2+bash3+bash4+bash5

#Covariances
fear6   ~~  bis2
fear2   ~~  bis2
fear2   ~~  fear6
fear5   ~~  bis1
bis3    ~~  bis4


host1   ~~  host3
host1   ~~  host5
host2   ~~  host3
host2   ~~  host6
host3   ~~  host5
host3   ~~  host6
host5   ~~  host6
reac1   ~~  host2
reac1   ~~  host3
reac1   ~~  reac3
reac5   ~~  reac6
reac2   ~~  reac3
reac4   ~~  host6
reac4   ~~  host5
reac4   ~~  host3
reac1   ~~  host6
reac6   ~~  host5
reac6   ~~  reac7
reac7   ~~  host4

bash1   ~~  bash2
bash4   ~~  bash5

"
```

```
aff.cfa<-cfa(aff.mod,data=dfs)

aff.sum<-summary(aff.cfa,fit.measures=TRUE,
standardized=TRUE)
```

```
lavaan 0.6-7 ended normally after 60 iterations

  Estimator                                         ML
  Optimization method                           NLMINB
  Number of free parameters                         81
                                                      
  Number of observations                           395
                                                      
Model Test User Model:
                                                      
  Test statistic                               674.591
  Degrees of freedom                               270
  P-value (Chi-square)                           0.000

Model Test Baseline Model:

  Test statistic                              5625.474
  Degrees of freedom                               325
  P-value                                        0.000

User Model versus Baseline Model:

  Comparative Fit Index (CFI)                    0.924
  Tucker-Lewis Index (TLI)                       0.908

Loglikelihood and Information Criteria:

  Loglikelihood user model (H0)             -12084.041
  Loglikelihood unrestricted model (H1)     -11746.745
                                                      
  Akaike (AIC)                               24330.081
  Bayesian (BIC)                             24652.371
  Sample-size adjusted Bayesian (BIC)        24395.358

Root Mean Square Error of Approximation:

  RMSEA                                          0.062
  90 Percent confidence interval - lower         0.056
  90 Percent confidence interval - upper         0.067
  P-value RMSEA <= 0.05                          0.001

Standardized Root Mean Square Residual:

  SRMR                                           0.067

Parameter Estimates:

  Standard errors                             Standard
  Information                                 Expected
  Information saturated (h1) model          Structured

Latent Variables:
                   Estimate  Std.Err  z-value  P(>|z|)   Std.lv  Std.all
  BIS =~                                                                
    fear2             1.000                               0.745    0.746
    fear5             1.081    0.067   16.041    0.000    0.805    0.806
    fear6             1.084    0.058   18.654    0.000    0.807    0.808
    bis1              0.632    0.070    9.050    0.000    0.471    0.471
    bis2              1.092    0.056   19.396    0.000    0.813    0.814
    bis3              1.108    0.068   16.318    0.000    0.825    0.826
    bis4              1.102    0.068   16.214    0.000    0.821    0.822
    bis5              1.029    0.068   15.231    0.000    0.766    0.767
  react =~                                                              
    reac2             1.000                               0.550    0.551
    reac1            -1.105    0.127   -8.671    0.000   -0.608   -0.609
    reac3             1.445    0.128   11.279    0.000    0.795    0.794
    reac4             1.010    0.120    8.416    0.000    0.556    0.556
    reac5             0.714    0.109    6.552    0.000    0.393    0.393
    reac6             0.756    0.110    6.888    0.000    0.416    0.419
    reac7             0.946    0.117    8.068    0.000    0.520    0.521
    host1             0.555    0.105    5.303    0.000    0.305    0.306
    host2             0.754    0.113    6.687    0.000    0.415    0.415
    host3             0.713    0.109    6.525    0.000    0.392    0.404
    host4             1.118    0.124    9.025    0.000    0.615    0.616
    host5             0.696    0.107    6.490    0.000    0.383    0.392
    host6             1.181    0.128    9.255    0.000    0.649    0.657
  BAS =~                                                                
    bash1             1.000                               0.765    0.766
    bash2             1.031    0.057   18.131    0.000    0.789    0.790
    bash3             0.836    0.072   11.563    0.000    0.640    0.641
    bash4             0.934    0.076   12.332    0.000    0.715    0.716
    bash5             0.947    0.076   12.496    0.000    0.725    0.726

Covariances:
                   Estimate  Std.Err  z-value  P(>|z|)   Std.lv  Std.all
 .fear6 ~~                                                              
   .bis2              0.161    0.025    6.458    0.000    0.161    0.471
 .fear2 ~~                                                              
   .bis2              0.113    0.025    4.433    0.000    0.113    0.292
   .fear6             0.096    0.025    3.790    0.000    0.096    0.245
 .fear5 ~~                                                              
   .bis1              0.057    0.030    1.924    0.054    0.057    0.110
 .bis3 ~~                                                               
   .bis4              0.046    0.022    2.078    0.038    0.046    0.145
 .host1 ~~                                                              
   .host3             0.142    0.039    3.650    0.000    0.142    0.168
   .host5             0.332    0.044    7.491    0.000    0.332    0.389
 .host2 ~~                                                              
   .host3             0.178    0.040    4.476    0.000    0.178    0.220
   .host6             0.125    0.034    3.732    0.000    0.125    0.185
 .host3 ~~                                                              
   .host5             0.263    0.041    6.414    0.000    0.263    0.330
   .host6             0.277    0.039    7.072    0.000    0.277    0.419
 .host5 ~~                                                              
   .host6             0.162    0.034    4.824    0.000    0.162    0.243
 .reac1 ~~                                                              
   .host2             0.162    0.041    3.955    0.000    0.162    0.225
   .host3             0.071    0.036    1.959    0.050    0.071    0.101
   .reac3             0.097    0.032    2.985    0.003    0.097    0.201
 .reac5 ~~                                                              
   .reac6             0.299    0.044    6.724    0.000    0.299    0.362
 .reac2 ~~                                                              
   .reac3             0.087    0.035    2.488    0.013    0.087    0.172
 .reac4 ~~                                                              
   .host6             0.252    0.038    6.588    0.000    0.252    0.408
   .host5             0.132    0.036    3.640    0.000    0.132    0.177
   .host3             0.181    0.039    4.600    0.000    0.181    0.245
 .reac1 ~~                                                              
   .host6             0.146    0.031    4.673    0.000    0.146    0.248
 .reac6 ~~                                                              
   .host5             0.147    0.034    4.394    0.000    0.147    0.182
   .reac7             0.091    0.036    2.523    0.012    0.091    0.118
 .reac7 ~~                                                              
   .host4             0.108    0.037    2.900    0.004    0.108    0.161
 .bash1 ~~                                                              
   .bash2             0.122    0.036    3.340    0.001    0.122    0.309
 .bash4 ~~                                                              
   .bash5             0.138    0.036    3.810    0.000    0.138    0.288
  BIS ~~                                                                
    react             0.291    0.039    7.497    0.000    0.709    0.709
    BAS              -0.338    0.043   -7.904    0.000   -0.593   -0.593
  react ~~                                                              
    BAS              -0.256    0.037   -6.926    0.000   -0.608   -0.608

Variances:
                   Estimate  Std.Err  z-value  P(>|z|)   Std.lv  Std.all
   .fear2             0.443    0.037   12.104    0.000    0.443    0.444
   .fear5             0.350    0.030   11.592    0.000    0.350    0.351
   .fear6             0.346    0.031   11.284    0.000    0.346    0.347
   .bis1              0.776    0.057   13.623    0.000    0.776    0.778
   .bis2              0.336    0.030   11.179    0.000    0.336    0.337
   .bis3              0.316    0.029   10.765    0.000    0.316    0.317
   .bis4              0.324    0.030   10.857    0.000    0.324    0.325
   .bis5              0.410    0.034   12.194    0.000    0.410    0.411
   .reac2             0.695    0.054   12.761    0.000    0.695    0.697
   .reac1             0.626    0.053   11.804    0.000    0.626    0.629
   .reac3             0.372    0.041    9.096    0.000    0.372    0.370
   .reac4             0.689    0.053   12.996    0.000    0.689    0.690
   .reac5             0.843    0.062   13.675    0.000    0.843    0.845
   .reac6             0.810    0.059   13.742    0.000    0.810    0.824
   .reac7             0.726    0.055   13.221    0.000    0.726    0.728
   .host1             0.904    0.065   13.844    0.000    0.904    0.907
   .host2             0.826    0.061   13.444    0.000    0.826    0.828
   .host3             0.788    0.058   13.664    0.000    0.788    0.837
   .host4             0.620    0.049   12.705    0.000    0.620    0.621
   .host5             0.807    0.058   13.988    0.000    0.807    0.846
   .host6             0.554    0.046   12.020    0.000    0.554    0.568
   .bash1             0.412    0.045    9.160    0.000    0.412    0.413
   .bash2             0.375    0.044    8.622    0.000    0.375    0.376
   .bash3             0.588    0.048   12.146    0.000    0.588    0.589
   .bash4             0.486    0.046   10.563    0.000    0.486    0.487
   .bash5             0.472    0.045   10.394    0.000    0.472    0.473
    BIS               0.555    0.066    8.371    0.000    1.000    1.000
    react             0.303    0.056    5.423    0.000    1.000    1.000
    BAS               0.586    0.073    8.016    0.000    1.000    1.000
```

```
aff.sum$PE%>%
filter(op!="~~")%>%
mutate(Parameter=paste(lhs,op,rhs))%>%
select(Parameter,b=est,SE=se,z,beta=std.all,p=pvalue)%>%
kable(digits=3)
```

| Parameter | b | SE | z | beta | p |
| --- | --- | --- | --- | --- | --- |
| BIS =~ fear2 | 1.000 | 0.000 | NA | 0.746 | NA |
| BIS =~ fear5 | 1.081 | 0.067 | 16.041 | 0.806 | 0 |
| BIS =~ fear6 | 1.084 | 0.058 | 18.654 | 0.808 | 0 |
| BIS =~ bis1 | 0.632 | 0.070 | 9.050 | 0.471 | 0 |
| BIS =~ bis2 | 1.092 | 0.056 | 19.396 | 0.814 | 0 |
| BIS =~ bis3 | 1.108 | 0.068 | 16.318 | 0.826 | 0 |
| BIS =~ bis4 | 1.102 | 0.068 | 16.214 | 0.822 | 0 |
| BIS =~ bis5 | 1.029 | 0.068 | 15.231 | 0.767 | 0 |
| react =~ reac2 | 1.000 | 0.000 | NA | 0.551 | NA |
| react =~ reac1 | -1.105 | 0.127 | -8.671 | -0.609 | 0 |
| react =~ reac3 | 1.445 | 0.128 | 11.279 | 0.794 | 0 |
| react =~ reac4 | 1.010 | 0.120 | 8.416 | 0.556 | 0 |
| react =~ reac5 | 0.714 | 0.109 | 6.552 | 0.393 | 0 |
| react =~ reac6 | 0.756 | 0.110 | 6.888 | 0.419 | 0 |
| react =~ reac7 | 0.946 | 0.117 | 8.068 | 0.521 | 0 |
| react =~ host1 | 0.555 | 0.105 | 5.303 | 0.306 | 0 |
| react =~ host2 | 0.754 | 0.113 | 6.687 | 0.415 | 0 |
| react =~ host3 | 0.713 | 0.109 | 6.525 | 0.404 | 0 |
| react =~ host4 | 1.118 | 0.124 | 9.025 | 0.616 | 0 |
| react =~ host5 | 0.696 | 0.107 | 6.490 | 0.392 | 0 |
| react =~ host6 | 1.181 | 0.128 | 9.255 | 0.657 | 0 |
| BAS =~ bash1 | 1.000 | 0.000 | NA | 0.766 | NA |
| BAS =~ bash2 | 1.031 | 0.057 | 18.131 | 0.790 | 0 |
| BAS =~ bash3 | 0.836 | 0.072 | 11.563 | 0.641 | 0 |
| BAS =~ bash4 | 0.934 | 0.076 | 12.332 | 0.716 | 0 |
| BAS =~ bash5 | 0.947 | 0.076 | 12.496 | 0.726 | 0 |

#ActionRatingsCFA

```
act.mod<-"
own_proj=~own_projects+doing_sports+creative
media=~information+digital_social_contacts+social_media
security=~washing_hands+disregard_curfew+stockpiling

#Covariance

digital_social_contacts ~~  washing_hands
doing_sports    ~~  digital_social_contacts
own_projects    ~~  digital_social_contacts
creative    ~~  digital_social_contacts
stockpiling~~washing_hands
disregard_curfew~~stockpiling
"
```

```
act.fit<-cfa(act.mod,data=dfs)

act.sum<-summary(act.fit,fit.measures=TRUE,
standardized=TRUE)
```

```
lavaan 0.6-7 ended normally after 64 iterations

  Estimator                                         ML
  Optimization method                           NLMINB
  Number of free parameters                         27
                                                      
  Number of observations                           395
                                                      
Model Test User Model:
                                                      
  Test statistic                                31.086
  Degrees of freedom                                18
  P-value (Chi-square)                           0.028

Model Test Baseline Model:

  Test statistic                               650.548
  Degrees of freedom                                36
  P-value                                        0.000

User Model versus Baseline Model:

  Comparative Fit Index (CFI)                    0.979
  Tucker-Lewis Index (TLI)                       0.957

Loglikelihood and Information Criteria:

  Loglikelihood user model (H0)              -4730.090
  Loglikelihood unrestricted model (H1)      -4714.547
                                                      
  Akaike (AIC)                                9514.180
  Bayesian (BIC)                              9621.610
  Sample-size adjusted Bayesian (BIC)         9535.938

Root Mean Square Error of Approximation:

  RMSEA                                          0.043
  90 Percent confidence interval - lower         0.014
  90 Percent confidence interval - upper         0.068
  P-value RMSEA <= 0.05                          0.649

Standardized Root Mean Square Residual:

  SRMR                                           0.040

Parameter Estimates:

  Standard errors                             Standard
  Information                                 Expected
  Information saturated (h1) model          Structured

Latent Variables:
                   Estimate  Std.Err  z-value  P(>|z|)   Std.lv  Std.all
  own_proj =~                                                           
    own_projects      1.000                               0.830    0.832
    doing_sports      0.697    0.075    9.341    0.000    0.578    0.580
    creative          0.875    0.087   10.072    0.000    0.726    0.727
  media =~                                                              
    information       1.000                               0.677    0.678
    dgtl_scl_cntct    0.343    0.089    3.849    0.000    0.232    0.233
    social_media      1.168    0.193    6.041    0.000    0.790    0.791
  security =~                                                           
    washing_hands     1.000                               0.959    0.964
    disregard_crfw   -0.309    0.271   -1.142    0.253   -0.296   -0.297
    stockpiling       1.249    0.438    2.850    0.004    1.198    1.199

Covariances:
                             Estimate  Std.Err  z-value  P(>|z|)   Std.lv
 .digital_social_contacts ~~                                             
   .washing_hands               0.218    0.045    4.841    0.000    0.218
 .doing_sports ~~                                                        
   .dgtl_scl_cntct              0.313    0.050    6.238    0.000    0.313
 .own_projects ~~                                                        
   .dgtl_scl_cntct              0.334    0.051    6.613    0.000    0.334
 .creative ~~                                                            
   .dgtl_scl_cntct              0.294    0.050    5.884    0.000    0.294
 .washing_hands ~~                                                       
   .stockpiling                -1.094    1.070   -1.023    0.307   -1.094
 .disregard_curfew ~~                                                    
   .stockpiling                 0.372    0.144    2.588    0.010    0.372
  own_proj ~~                                                            
    media                      -0.053    0.038   -1.406    0.160   -0.095
    security                    0.034    0.030    1.150    0.250    0.043
  media ~~                                                               
    security                    0.137    0.042    3.232    0.001    0.211
  Std.all
         
    0.854
         
    0.399
         
    0.625
         
    0.444
         
   -6.264
         
    0.590
         
   -0.095
    0.043
         
    0.211

Variances:
                   Estimate  Std.Err  z-value  P(>|z|)   Std.lv  Std.all
   .own_projects      0.306    0.063    4.832    0.000    0.306    0.308
   .doing_sports      0.661    0.055   11.945    0.000    0.661    0.664
   .creative          0.468    0.056    8.298    0.000    0.468    0.471
   .information       0.540    0.082    6.613    0.000    0.540    0.541
   .dgtl_scl_cntct    0.936    0.068   13.837    0.000    0.936    0.946
   .social_media      0.373    0.102    3.671    0.000    0.373    0.374
   .washing_hands     0.070    0.793    0.088    0.930    0.070    0.070
   .disregard_crfw    0.910    0.099    9.149    0.000    0.910    0.912
   .stockpiling      -0.438    1.582   -0.277    0.782   -0.438   -0.439
    own_proj          0.688    0.090    7.664    0.000    1.000    1.000
    media             0.458    0.094    4.896    0.000    1.000    1.000
    security          0.920    0.796    1.156    0.248    1.000    1.000
```

DefenseMeasuresIntercorrelations

```
df%>%
mutate(curfew_rec=7-disregard_curfew)%>%
rowwise()%>%
transmute(projects=mean(own_projects,doing_sports,creative,na.rm=T),
media=mean(information,digital_social_contacts,social_media,na.rm=T),
security=mean(washing_hands,curfew_rec,stockpiling,na.rm=T),
sysj=sysjust)%>%
corr.test(method="pearson")
```

```
Call:corr.test(x = ., method = "pearson")
Correlation matrix 
         projects media security  sysj
projects     1.00 -0.08     0.03  0.07
media       -0.08  1.00     0.09 -0.04
security     0.03  0.09     1.00  0.10
sysj         0.07 -0.04     0.10  1.00
Sample Size 
[1] 395
Probability values (Entries above the diagonal are adjusted for multiple tests.) 
         projects media security sysj
projects     0.00  0.50     0.77 0.50
media        0.12  0.00     0.32 0.77
security     0.49  0.06     0.00 0.25
sysj         0.14  0.38     0.04 0.00

 To see confidence intervals of the correlations, print with the short=FALSE option
```

```
(df%>%
mutate(curfew_rec=7-disregard_curfew)%>%
rowwise()%>%
transmute(projects=mean(own_projects,doing_sports,creative,na.rm=T),
media=mean(information,digital_social_contacts,social_media,na.rm=T),
security=mean(washing_hands,curfew_rec,stockpiling,na.rm=T),
sysj=sysjust)%>%
ungroup()%>%
summarize(projects_m=mean(projects),
projects_sd=sd(projects),
media_m=mean(media),
media_sd=sd(media),
security_m=mean(security),
security_sd=sd(security),
sysjust_m=mean(sysj),
sysjust_df=sd(sysj)))
```

```
# A tibble: 1 x 8
  projects_m projects_sd media_m media_sd security_m security_sd sysjust_m
       <dbl>       <dbl>   <dbl>    <dbl>      <dbl>       <dbl>     <dbl>
1       3.98       0.768    2.42    0.897       3.94       0.901      4.72
# ... with 1 more variable: sysjust_df <dbl>
```

##SJCFA

```
sj.mod<-"
sysj=~SJ1+SJ2+SJ3+SJ4+SJ5+SJ6+SJ7+SJ8

#Cov
SJ2 ~~  SJ5
SJ2 ~~  SJ4
SJ6 ~~  SJ7
SJ3 ~~  SJ6 
SJ4 ~~  SJ5
SJ7 ~~  SJ8
SJ1 ~~  SJ8
SJ5 ~~  SJ6
#SJ2    ~~  SJ6
"
```

```
sj.fit<-cfa(sj.mod,data=df)

sj.sum<-summary(sj.fit,fit.measures=T,
standardized=T,modindices=T)
```

```
lavaan 0.6-7 ended normally after 38 iterations

  Estimator                                         ML
  Optimization method                           NLMINB
  Number of free parameters                         24
                                                      
  Number of observations                           395
                                                      
Model Test User Model:
                                                      
  Test statistic                                30.458
  Degrees of freedom                                12
  P-value (Chi-square)                           0.002

Model Test Baseline Model:

  Test statistic                               692.604
  Degrees of freedom                                28
  P-value                                        0.000

User Model versus Baseline Model:

  Comparative Fit Index (CFI)                    0.972
  Tucker-Lewis Index (TLI)                       0.935

Loglikelihood and Information Criteria:

  Loglikelihood user model (H0)              -5538.705
  Loglikelihood unrestricted model (H1)      -5523.476
                                                      
  Akaike (AIC)                               11125.411
  Bayesian (BIC)                             11220.904
  Sample-size adjusted Bayesian (BIC)        11144.752

Root Mean Square Error of Approximation:

  RMSEA                                          0.062
  90 Percent confidence interval - lower         0.035
  90 Percent confidence interval - upper         0.090
  P-value RMSEA <= 0.05                          0.204

Standardized Root Mean Square Residual:

  SRMR                                           0.036

Parameter Estimates:

  Standard errors                             Standard
  Information                                 Expected
  Information saturated (h1) model          Structured

Latent Variables:
                   Estimate  Std.Err  z-value  P(>|z|)   Std.lv  Std.all
  sysj =~                                                               
    SJ1               1.000                               1.166    0.737
    SJ2               0.621    0.072    8.597    0.000    0.724    0.478
    SJ3              -0.907    0.099   -9.203    0.000   -1.058   -0.578
    SJ4               0.479    0.070    6.843    0.000    0.558    0.379
    SJ5               0.269    0.061    4.387    0.000    0.314    0.253
    SJ6               0.808    0.104    7.744    0.000    0.942    0.560
    SJ7              -0.896    0.091   -9.801    0.000   -1.044   -0.664
    SJ8               0.666    0.078    8.559    0.000    0.776    0.498

Covariances:
                   Estimate  Std.Err  z-value  P(>|z|)   Std.lv  Std.all
 .SJ2 ~~                                                                
   .SJ5               0.519    0.087    5.951    0.000    0.519    0.324
   .SJ4               0.551    0.101    5.436    0.000    0.551    0.303
 .SJ6 ~~                                                                
   .SJ7               0.620    0.127    4.889    0.000    0.620    0.378
 .SJ3 ~~                                                                
   .SJ6               0.649    0.144    4.499    0.000    0.649    0.312
 .SJ4 ~~                                                                
   .SJ5               0.395    0.086    4.618    0.000    0.395    0.242
 .SJ7 ~~                                                                
   .SJ8               0.357    0.112    3.204    0.001    0.357    0.225
 .SJ1 ~~                                                                
   .SJ8               0.167    0.116    1.437    0.151    0.167    0.116
 .SJ5 ~~                                                                
   .SJ6               0.288    0.092    3.140    0.002    0.288    0.172

Variances:
                   Estimate  Std.Err  z-value  P(>|z|)   Std.lv  Std.all
   .SJ1               1.142    0.130    8.770    0.000    1.142    0.457
   .SJ2               1.776    0.135   13.200    0.000    1.776    0.772
   .SJ3               2.227    0.191   11.662    0.000    2.227    0.666
   .SJ4               1.862    0.137   13.579    0.000    1.862    0.857
   .SJ5               1.439    0.104   13.829    0.000    1.439    0.936
   .SJ6               1.945    0.182   10.671    0.000    1.945    0.687
   .SJ7               1.385    0.141    9.816    0.000    1.385    0.559
   .SJ8               1.828    0.158   11.553    0.000    1.828    0.752
    sysj              1.360    0.188    7.222    0.000    1.000    1.000

Modification Indices:

   lhs op rhs     mi    epc sepc.lv sepc.all sepc.nox
26 SJ1 ~~ SJ2  0.213  0.038   0.038    0.027    0.027
27 SJ1 ~~ SJ3  3.296 -0.252  -0.252   -0.158   -0.158
28 SJ1 ~~ SJ4  0.009 -0.007  -0.007   -0.005   -0.005
29 SJ1 ~~ SJ5  0.117  0.025   0.025    0.020    0.020
30 SJ1 ~~ SJ6 13.156 -0.555  -0.555   -0.372   -0.372
31 SJ1 ~~ SJ7  0.294 -0.085  -0.085   -0.067   -0.067
32 SJ2 ~~ SJ3  2.482  0.168   0.168    0.085    0.085
33 SJ2 ~~ SJ6 12.777  0.416   0.416    0.224    0.224
34 SJ2 ~~ SJ7  0.095 -0.029  -0.029   -0.019   -0.019
35 SJ2 ~~ SJ8  7.716 -0.257  -0.257   -0.143   -0.143
36 SJ3 ~~ SJ4  0.001 -0.003  -0.003   -0.002   -0.002
37 SJ3 ~~ SJ5  0.371  0.056   0.056    0.031    0.031
38 SJ3 ~~ SJ7  0.070 -0.039  -0.039   -0.022   -0.022
39 SJ3 ~~ SJ8  0.024  0.023   0.023    0.011    0.011
40 SJ4 ~~ SJ6  0.499 -0.077  -0.077   -0.041   -0.041
41 SJ4 ~~ SJ7  0.779  0.083   0.083    0.052    0.052
42 SJ4 ~~ SJ8  4.078  0.187   0.187    0.101    0.101
43 SJ5 ~~ SJ7  0.032 -0.014  -0.014   -0.010   -0.010
44 SJ5 ~~ SJ8  0.006 -0.006  -0.006   -0.004   -0.004
45 SJ6 ~~ SJ8  2.423  0.209   0.209    0.111    0.111
```

```
sj.sum$MI%>%
arrange(-mi)
```

```
   lhs op rhs     mi    epc sepc.lv sepc.all sepc.nox
1  SJ1 ~~ SJ6 13.156 -0.555  -0.555   -0.372   -0.372
2  SJ2 ~~ SJ6 12.777  0.416   0.416    0.224    0.224
3  SJ2 ~~ SJ8  7.716 -0.257  -0.257   -0.143   -0.143
4  SJ4 ~~ SJ8  4.078  0.187   0.187    0.101    0.101
5  SJ1 ~~ SJ3  3.296 -0.252  -0.252   -0.158   -0.158
6  SJ2 ~~ SJ3  2.482  0.168   0.168    0.085    0.085
7  SJ6 ~~ SJ8  2.423  0.209   0.209    0.111    0.111
8  SJ4 ~~ SJ7  0.779  0.083   0.083    0.052    0.052
9  SJ4 ~~ SJ6  0.499 -0.077  -0.077   -0.041   -0.041
10 SJ3 ~~ SJ5  0.371  0.056   0.056    0.031    0.031
11 SJ1 ~~ SJ7  0.294 -0.085  -0.085   -0.067   -0.067
12 SJ1 ~~ SJ2  0.213  0.038   0.038    0.027    0.027
13 SJ1 ~~ SJ5  0.117  0.025   0.025    0.020    0.020
14 SJ2 ~~ SJ7  0.095 -0.029  -0.029   -0.019   -0.019
15 SJ3 ~~ SJ7  0.070 -0.039  -0.039   -0.022   -0.022
16 SJ5 ~~ SJ7  0.032 -0.014  -0.014   -0.010   -0.010
17 SJ3 ~~ SJ8  0.024  0.023   0.023    0.011    0.011
18 SJ1 ~~ SJ4  0.009 -0.007  -0.007   -0.005   -0.005
19 SJ5 ~~ SJ8  0.006 -0.006  -0.006   -0.004   -0.004
20 SJ3 ~~ SJ4  0.001 -0.003  -0.003   -0.002   -0.002
```

##LonelinessCFA

```
lon.mod<-"
lonel=~lsoc5+lsoc1+lsoc2+lsoc4+lsoc3+
lfam1+lfam2+lfam3+lfam4+lfam5+
lrom1+lrom2+lrom3+lrom4+lrom5

#Covariances
#lfam3  ~~  lfam4
#lsoc3  ~~  lsoc5
#lsoc2  ~~  lsoc4
#lsoc1  ~~  lsoc4
#lsoc1  ~~  lsoc2
##lfam1 ~~  lfam2
#lfam1  ~~  lfam3
#lfam3  ~~  lfam5
#lfam4  ~~  lfam5
#lrom1  ~~  lrom4   
#lrom1  ~~  lrom3
#lrom2  ~~  lrom3
#lrom3  ~~  lrom5
#lrom3  ~~  lrom4
#lsoc5  ~~  lfam2
#lsoc3  ~~  lfam2   
#lfam4  ~~  lrom4
#lsoc5  ~~  lfam1
#lsoc3  ~~  lfam1
#lsoc3  ~~  lrom3
#lfam1  ~~  lrom4
#lsoc1  ~~  lrom1
#lrom2  ~~  lrom4
#lrom1  ~~  lrom2
#lsoc2  ~~  lfam2
#lsoc1  ~~  lfam2
#lsoc1  ~~  lfam1
#lsoc2  ~~  lfam1
#lsoc4  ~~  lfam2

lrom1~~lrom2
lrom1~~lrom3
lrom1~~lrom4
lrom1~~lrom5
lrom2~~lrom3
lrom2~~lrom4
lrom2~~lrom5
lrom3~~lrom4
lrom3~~lrom5
lrom4~~lrom5

lsoc1~~lsoc2
lsoc1~~lsoc3
lsoc1~~lsoc4
lsoc1~~lsoc5
lsoc2~~lsoc3
lsoc2~~lsoc4
lsoc2~~lsoc5
lsoc3~~lsoc4
lsoc3~~lsoc5
lsoc4~~lsoc5

lfam1~~lfam2
lfam1~~lfam3
lfam1~~lfam4
lfam1~~lfam5
lfam2~~lfam3
lfam2~~lfam4
lfam2~~lfam5
lfam3~~lfam4
lfam3~~lfam5
lfam4~~lfam5
"
```

```
lon.fit<-cfa(lon.mod,data=df)

lon.sum<-summary(lon.fit,fit.measures=T,
standardized=T)
```

```
lavaan 0.6-7 ended normally after 101 iterations

  Estimator                                         ML
  Optimization method                           NLMINB
  Number of free parameters                         60
                                                      
  Number of observations                           395
                                                      
Model Test User Model:
                                                      
  Test statistic                                94.727
  Degrees of freedom                                60
  P-value (Chi-square)                           0.003

Model Test Baseline Model:

  Test statistic                              4285.133
  Degrees of freedom                               105
  P-value                                        0.000

User Model versus Baseline Model:

  Comparative Fit Index (CFI)                    0.992
  Tucker-Lewis Index (TLI)                       0.985

Loglikelihood and Information Criteria:

  Loglikelihood user model (H0)              -8721.417
  Loglikelihood unrestricted model (H1)      -8674.054
                                                      
  Akaike (AIC)                               17562.835
  Bayesian (BIC)                             17801.568
  Sample-size adjusted Bayesian (BIC)        17611.188

Root Mean Square Error of Approximation:

  RMSEA                                          0.038
  90 Percent confidence interval - lower         0.023
  90 Percent confidence interval - upper         0.052
  P-value RMSEA <= 0.05                          0.910

Standardized Root Mean Square Residual:

  SRMR                                           0.037

Parameter Estimates:

  Standard errors                             Standard
  Information                                 Expected
  Information saturated (h1) model          Structured

Latent Variables:
                   Estimate  Std.Err  z-value  P(>|z|)   Std.lv  Std.all
  lonel =~                                                              
    lsoc5             1.000                               0.784    0.673
    lsoc1            -1.041    0.118   -8.823    0.000   -0.816   -0.607
    lsoc2            -0.874    0.097   -8.971    0.000   -0.685   -0.630
    lsoc4            -0.854    0.095   -8.983    0.000   -0.669   -0.620
    lsoc3             0.897    0.082   10.954    0.000    0.703    0.627
    lfam1             0.801    0.178    4.509    0.000    0.627    0.443
    lfam2             0.712    0.149    4.772    0.000    0.558    0.517
    lfam3            -0.859    0.181   -4.756    0.000   -0.673   -0.511
    lfam4            -0.816    0.170   -4.810    0.000   -0.640   -0.529
    lfam5            -0.764    0.168   -4.550    0.000   -0.599   -0.453
    lrom1            -0.816    0.222   -3.669    0.000   -0.639   -0.259
    lrom2            -0.798    0.220   -3.628    0.000   -0.625   -0.255
    lrom3             1.046    0.222    4.723    0.000    0.820    0.383
    lrom4            -0.763    0.215   -3.545    0.000   -0.598   -0.247
    lrom5             0.930    0.211    4.403    0.000    0.729    0.340

Covariances:
                   Estimate  Std.Err  z-value  P(>|z|)   Std.lv  Std.all
 .lrom1 ~~                                                              
   .lrom2             5.192    0.400   12.973    0.000    5.192    0.918
   .lrom3            -2.639    0.292   -9.037    0.000   -2.639   -0.560
   .lrom4             5.224    0.398   13.119    0.000    5.224    0.935
   .lrom5            -3.162    0.307  -10.298    0.000   -3.162   -0.657
 .lrom2 ~~                                                              
   .lrom3            -2.562    0.289   -8.877    0.000   -2.562   -0.547
   .lrom4             5.096    0.392   12.984    0.000    5.096    0.917
   .lrom5            -3.110    0.304  -10.225    0.000   -3.110   -0.650
 .lrom3 ~~                                                              
   .lrom4            -2.452    0.283   -8.672    0.000   -2.452   -0.529
   .lrom5             2.698    0.266   10.145    0.000    2.698    0.677
 .lrom4 ~~                                                              
   .lrom5            -3.130    0.302  -10.376    0.000   -3.130   -0.662
 .lsoc1 ~~                                                              
   .lsoc2             0.129    0.117    1.107    0.268    0.129    0.144
   .lsoc3            -0.151    0.121   -1.250    0.211   -0.151   -0.162
   .lsoc4             0.263    0.118    2.231    0.026    0.263    0.290
 .lsoc5 ~~                                                              
   .lsoc1            -0.212    0.131   -1.614    0.106   -0.212   -0.231
 .lsoc2 ~~                                                              
   .lsoc3            -0.169    0.100   -1.683    0.092   -0.169   -0.230
   .lsoc4             0.215    0.097    2.216    0.027    0.215    0.302
 .lsoc5 ~~                                                              
   .lsoc2            -0.145    0.108   -1.343    0.179   -0.145   -0.200
 .lsoc4 ~~                                                              
   .lsoc3            -0.187    0.099   -1.887    0.059   -0.187   -0.253
 .lsoc5 ~~                                                              
   .lsoc3             0.354    0.115    3.067    0.002    0.354    0.471
   .lsoc4            -0.165    0.107   -1.548    0.122   -0.165   -0.227
 .lfam1 ~~                                                              
   .lfam2             0.311    0.090    3.448    0.001    0.311    0.264
   .lfam3            -0.729    0.116   -6.296    0.000   -0.729   -0.506
   .lfam4            -0.496    0.104   -4.775    0.000   -0.496   -0.381
   .lfam5            -0.468    0.108   -4.338    0.000   -0.468   -0.312
 .lfam2 ~~                                                              
   .lfam3            -0.368    0.089   -4.121    0.000   -0.368   -0.351
   .lfam4            -0.374    0.084   -4.471    0.000   -0.374   -0.394
   .lfam5            -0.453    0.087   -5.182    0.000   -0.453   -0.415
 .lfam3 ~~                                                              
   .lfam4             0.757    0.108    7.036    0.000    0.757    0.652
   .lfam5             0.689    0.109    6.326    0.000    0.689    0.516
 .lfam4 ~~                                                              
   .lfam5             0.525    0.099    5.316    0.000    0.525    0.434

Variances:
                   Estimate  Std.Err  z-value  P(>|z|)   Std.lv  Std.all
   .lsoc5             0.741    0.135    5.508    0.000    0.741    0.547
   .lsoc1             1.140    0.166    6.884    0.000    1.140    0.631
   .lsoc2             0.711    0.111    6.395    0.000    0.711    0.602
   .lsoc4             0.716    0.108    6.608    0.000    0.716    0.615
   .lsoc3             0.763    0.118    6.462    0.000    0.763    0.606
   .lfam1             1.616    0.143   11.313    0.000    1.616    0.804
   .lfam2             0.856    0.087    9.887    0.000    0.856    0.733
   .lfam3             1.280    0.128    9.999    0.000    1.280    0.739
   .lfam4             1.052    0.109    9.618    0.000    1.052    0.720
   .lfam5             1.390    0.125   11.135    0.000    1.390    0.795
   .lrom1             5.683    0.419   13.575    0.000    5.683    0.933
   .lrom2             5.625    0.414   13.591    0.000    5.625    0.935
   .lrom3             3.903    0.305   12.814    0.000    3.903    0.853
   .lrom4             5.494    0.403   13.623    0.000    5.494    0.939
   .lrom5             4.072    0.310   13.140    0.000    4.072    0.885
    lonel             0.614    0.148    4.155    0.000    1.000    1.000
```

```
df<- df %>%
rowwise() %>%
mutate(media_use = mean(information, social_media, gluedto_tv))
```

# Additional analyses (as requested by reviewers)

```
# 


df <- df %>%
  mutate(edu_fct = fct_recode(education,
                              "bis FH-Reife / Lehrabschluss" = "bn1",
                              "bis FH-Reife / Lehrabschluss" = "bn2",
                              "bis FH-Reife / Lehrabschluss" = "bn3",
                              "bis FH-Reife / Lehrabschluss" = "bn4",
                              "bis FH-Reife / Lehrabschluss" = "bn5",
                              "bis FH-Reife / Lehrabschluss" = "bn7",
                              "allg. HS-Reife" = "bn6",
                              "HS-Abschluss" = "bn8",
                              "HS-Abschluss" = "bn9",
                              "HS-Abschluss" = "bn10",
                              "HS-Abschluss" = "-oth-")) %>% 
  mutate(edu_fct = fct_relevel(edu_fct, "HS-Abschluss", after = Inf)) %>% 
  mutate(edu_degree = case_when(
    edu_fct == "allg. HS-Reife" ~ 0,
    edu_fct == "bis FH-Reife / Lehrabschluss"  ~ 0,
    edu_fct == "HS-Abschluss" ~ 1
  ))

# count(df,  edu_degree)
```

#Model

The final SEM includes Discrepancy and Expectancy Violation as predictors for Affect. The assumed affect mediators are BIS, BAS, and reactance affect: all facets of motivational-affective states relevant for threat-defenses.

Loneliness is a temporally stable set of circumstances we also assume to predict defensive strategies.

As defenses, we used four latent variables: Own Projects, Media Use, Security-enhacing efforts, and System Justification.

Direct and indirect effects will be reported. We assume that discrepancies influence BIS, BAS, and reactance, which in turn will predict preferences for concrete strategies: - own projects are idiosynchratic and related to personal growth; they should be predicted by BAS-related approach affect and determination - Use of (social) media is a pro-active way of (re-)establishing social contacts, and should also be related to BAS-state - reactance is a feeling of hostility and being treated injustly; it should relate to both increased likelihood to disregard security efforts and instructions; and predict lower system justification.

---

# SEM with Covariates

Including significant correlations from the correlation table, include covariates for the respective latent variables. We have included riskgroup, edu\_degree, nationality (Austria), gender, and age as covariates.

```
df <- df %>% 
  mutate(sex = recode(sex, "male" = 0, "female" = 1),
         country = recode(country, "DE" = 0, "AT" = 1))
```

```
sem.cov.mod<-"
#LatentVariables

own_proj=~own_projects+doing_sports+creative
media=~digital_social_contacts+social_media
security=~washing_hands+disregard_curfew+stockpiling+information

sysj=~SJ1+SJ2+SJ3+SJ4+SJ5+SJ6+SJ7+SJ8


lonel=~lsoc5+lsoc1+lsoc2+lsoc4+lsoc3+
lfam1+lfam2+lfam3+lfam4+lfam5+
lrom1+lrom2+lrom3+lrom4+lrom5

discr=~aut2+aut1+aut3+aut4+
uncer1+uncer2+uncer3+uncer4+
agenc1+agenc2+agenc3+agenc4

exp=~expec1+expec2+expec3

BIS=~fear2+fear5+fear6+bis1+bis2+bis3+bis4+bis5
react=~reac2+reac1+reac3+reac4+reac5+reac6+reac7+host1+host2+host3+host4+host5+host6
BAS=~bash1+bash2+bash3+bash4+bash5

#Regressions

BIS~a1*discr
BAS~a2*discr
react~a3*discr

BIS~e1*exp
BAS~e2*exp
react~e3*exp


own_proj~b1.1*BIS
own_proj~b2.1*BAS
own_proj~b3.1*react
own_proj~l1*lonel

media~b1.2*BIS
media~b2.2*BAS
media~b3.2*react
media~l2*lonel


security~b1.3*BIS
security~b2.3*BAS
security~b3.3*react
security~l3*lonel


sysj~b1.4*BIS
sysj~b2.4*BAS
sysj~b3.4*react
sysj~l4*lonel

# Covariates
BIS ~ sex
media ~ sex
security ~ sex

BIS ~ risk
own_proj ~ risk
sysj ~ risk

BAS ~ edu_degree
react~ edu_degree

BAS ~ age
react ~ age
own_proj ~ age

own_proj ~ country


#Directeffects

own_proj~cd1*discr
media~cd2*discr
security~cd3*discr
sysj~cd4*discr

own_proj~ce1*exp
media~ce2*exp
security~ce3*exp
sysj~ce4*exp


#indirecteffects-discrepancy
ab1.1:=a1*b1.1
ab1.2:=a1*b1.2
ab1.3:=a1*b1.3
ab1.4:=a1*b1.4

ab2.1:=a2*b2.1
ab2.2:=a2*b2.2
ab2.3:=a2*b2.3
ab2.4:=a2*b2.4

ab3.1:=a3*b3.1
ab3.2:=a3*b3.2
ab3.3:=a3*b3.3
ab3.4:=a3*b3.4


#indirecteffects-expectancy
eb1.1:=e1*b1.1
eb1.2:=e1*b1.2
eb1.3:=e1*b1.3
eb1.4:=e1*b1.4

eb2.1:=e2*b2.1
eb2.2:=e2*b2.2
eb2.3:=e2*b2.3
eb2.4:=e2*b2.4

eb3.1:=e3*b3.1
eb3.2:=e3*b3.2
eb3.3:=e3*b3.3
eb3.4:=e3*b3.4


#totaleffects-discrepancy

tot.d1.1:=cd1+ab1.1
tot.d1.2:=cd2+ab1.2
tot.d1.3:=cd3+ab1.3
#tot.d1.4:=cd4+ab1.4

tot.d2.1:=cd1+ab2.1
tot.d2.2:=cd2+ab2.2
tot.d2.3:=cd3+ab2.3
#tot.d2.4:=cd4+ab2.4

tot.d3.1:=cd1+ab3.1
tot.d3.2:=cd2+ab3.2
tot.d3.3:=cd3+ab3.3

#Covariances
aut1~~aut2
aut1~~aut3
aut1~~aut4
aut2~~aut3
aut2~~aut4
aut3~~aut4

uncer1~~uncer2
uncer1~~uncer3
uncer1~~uncer4
uncer2~~uncer3
uncer2~~uncer4
uncer3~~uncer4

agenc1~~agenc2
agenc1~~agenc3
agenc1~~agenc4
agenc2~~agenc3
agenc2~~agenc4
agenc3~~agenc4

reac2   ~~  reac3
reac5   ~~  reac6
reac4   ~~  host6
reac1   ~~  host6
reac6   ~~  host5
reac4   ~~  host3
reac4   ~~  host5
host1~~host5
host1   ~~  host3
host2   ~~  host3
host2   ~~  host5
host1   ~~  host2
host2   ~~  host6
host3   ~~  host5
host3   ~~  host6
host5   ~~  host6

fear6   ~~  bis2
fear2   ~~  bis2
fear2   ~~  fear6
reac4   ~~  host2
bis3    ~~  bis4

bash1~~bash2
bash4   ~~  bash5

SJ2~~SJ5
SJ2~~SJ4
SJ4~~SJ5
SJ5 ~~SJ6
SJ2~~SJ6
SJ7~~SJ8
SJ6~~SJ7
SJ3 ~~SJ6
SJ6 ~~SJ8
SJ4 ~~  SJ6
SJ4 ~~  SJ8

lrom1~~lrom2
lrom1~~lrom3
lrom1~~lrom4
lrom1~~lrom5
lrom2~~lrom3
lrom2~~lrom4
lrom2~~lrom5
lrom3~~lrom4
lrom3~~lrom5
lrom4~~lrom5

lsoc1~~lsoc2
lsoc1~~lsoc3
lsoc1~~lsoc4
lsoc1~~lsoc5
lsoc2~~lsoc3
lsoc2~~lsoc4
lsoc2~~lsoc5
lsoc3~~lsoc4
lsoc3~~lsoc5
lsoc4~~lsoc5

lfam1~~lfam2
lfam1~~lfam3
lfam1~~lfam4
lfam1~~lfam5
lfam2~~lfam3
lfam2~~lfam4
lfam2~~lfam5
lfam3~~lfam4
lfam3~~lfam5
lfam4~~lfam5

reac1   ~~  reac7


creative    ~~  social_media
own_projects    ~~  creative
information ~~  social_media

digital_social_contacts ~~  washing_hands
doing_sports    ~~  digital_social_contacts
own_projects    ~~  digital_social_contacts
creative    ~~  digital_social_contacts
stockpiling~~washing_hands
disregard_curfew~~stockpiling

BIS ~~  react

"
```

Fit

```
set.seed(92375)

tic("bootstrap cov")
sem.cov.boot.seed <- sem(sem.cov.mod, data = df, se = "bootstrap", bootstrap = 1000, parameterization = "theta")
toc()
```

```
bootstrap cov: 3870.48 sec elapsed
```

```
boot.cov.seed.sum <- summary(sem.cov.boot.seed, fit.measures=T)
```

```
lavaan 0.6-7 ended normally after 193 iterations

  Estimator                                         ML
  Optimization method                           NLMINB
  Number of free parameters                        290
                                                      
  Number of observations                           395
                                                      
Model Test User Model:
                                                      
  Test statistic                              4642.077
  Degrees of freedom                              2776
  P-value (Chi-square)                           0.000

Model Test Baseline Model:

  Test statistic                             17239.179
  Degrees of freedom                              2993
  P-value                                        0.000

User Model versus Baseline Model:

  Comparative Fit Index (CFI)                    0.869
  Tucker-Lewis Index (TLI)                       0.859

Loglikelihood and Information Criteria:

  Loglikelihood user model (H0)             -36911.405
  Loglikelihood unrestricted model (H1)     -34590.366
                                                      
  Akaike (AIC)                               74402.809
  Bayesian (BIC)                             75556.686
  Sample-size adjusted Bayesian (BIC)        74636.515

Root Mean Square Error of Approximation:

  RMSEA                                          0.041
  90 Percent confidence interval - lower         0.039
  90 Percent confidence interval - upper         0.043
  P-value RMSEA <= 0.05                          1.000

Standardized Root Mean Square Residual:

  SRMR                                           0.067

Parameter Estimates:

  Standard errors                            Bootstrap
  Number of requested bootstrap draws             1000
  Number of successful bootstrap draws             881

Latent Variables:
                   Estimate  Std.Err  z-value  P(>|z|)
  own_proj =~                                         
    own_projects      1.000                           
    doing_sports      1.353    0.424    3.194    0.001
    creative          1.005    0.111    9.083    0.000
  media =~                                            
    dgtl_scl_cntct    1.000                           
    social_media      0.407    0.471    0.863    0.388
  security =~                                         
    washing_hands     1.000                           
    disregard_crfw   -0.918    0.765   -1.199    0.230
    stockpiling       0.572    0.385    1.488    0.137
    information       0.213    0.239    0.888    0.375
  sysj =~                                             
    SJ1               1.000                           
    SJ2               0.506    0.083    6.058    0.000
    SJ3              -0.789    0.080   -9.830    0.000
    SJ4               0.404    0.084    4.780    0.000
    SJ5               0.226    0.070    3.213    0.001
    SJ6               0.545    0.098    5.562    0.000
    SJ7              -0.789    0.081   -9.729    0.000
    SJ8               0.604    0.083    7.246    0.000
  lonel =~                                            
    lsoc5             1.000                           
    lsoc1            -1.029    0.182   -5.667    0.000
    lsoc2            -0.906    0.133   -6.821    0.000
    lsoc4            -0.864    0.139   -6.210    0.000
    lsoc3             0.960    0.158    6.071    0.000
    lfam1             1.231    0.490    2.513    0.012
    lfam2             0.960    0.268    3.579    0.000
    lfam3            -1.136    0.305   -3.718    0.000
    lfam4            -1.019    0.253   -4.028    0.000
    lfam5            -0.971    0.280   -3.468    0.001
    lrom1            -1.143    0.503   -2.272    0.023
    lrom2            -1.151    0.525   -2.194    0.028
    lrom3             1.421    0.554    2.566    0.010
    lrom4            -1.072    0.481   -2.228    0.026
    lrom5             1.276    0.526    2.425    0.015
  discr =~                                            
    aut2              1.000                           
    aut1             -1.222    0.137   -8.893    0.000
    aut3              1.259    0.179    7.038    0.000
    aut4              0.790    0.108    7.318    0.000
    uncer1           -1.527    0.244   -6.266    0.000
    uncer2            1.108    0.180    6.158    0.000
    uncer3            0.895    0.176    5.079    0.000
    uncer4            2.033    0.255    7.964    0.000
    agenc1            1.376    0.208    6.612    0.000
    agenc2            1.239    0.207    6.001    0.000
    agenc3           -1.401    0.230   -6.082    0.000
    agenc4           -1.211    0.203   -5.977    0.000
  exp =~                                              
    expec1            1.000                           
    expec2           -1.055    0.094  -11.266    0.000
    expec3           -0.921    0.088  -10.494    0.000
  BIS =~                                              
    fear2             1.000                           
    fear5             1.263    0.090   13.971    0.000
    fear6             1.147    0.062   18.362    0.000
    bis1              0.771    0.091    8.510    0.000
    bis2              1.156    0.060   19.169    0.000
    bis3              1.180    0.067   17.537    0.000
    bis4              1.250    0.085   14.715    0.000
    bis5              1.113    0.071   15.747    0.000
  react =~                                            
    reac2             1.000                           
    reac1            -0.940    0.078  -12.035    0.000
    reac3             1.337    0.130   10.321    0.000
    reac4             0.864    0.144    6.005    0.000
    reac5             0.501    0.108    4.654    0.000
    reac6             0.562    0.128    4.396    0.000
    reac7             1.140    0.134    8.489    0.000
    host1             0.308    0.091    3.372    0.001
    host2             0.292    0.081    3.588    0.000
    host3             0.320    0.100    3.211    0.001
    host4             1.025    0.132    7.765    0.000
    host5             0.409    0.117    3.492    0.000
    host6             0.883    0.149    5.907    0.000
  BAS =~                                              
    bash1             1.000                           
    bash2             1.033    0.056   18.374    0.000
    bash3             0.854    0.087    9.782    0.000
    bash4             1.013    0.092   11.032    0.000
    bash5             0.997    0.085   11.712    0.000

Regressions:
                   Estimate  Std.Err  z-value  P(>|z|)
  BIS ~                                               
    discr     (a1)    1.384    0.218    6.349    0.000
  BAS ~                                               
    discr     (a2)   -1.238    0.196   -6.327    0.000
  react ~                                             
    discr     (a3)    1.206    0.172    7.030    0.000
  BIS ~                                               
    exp       (e1)    0.024    0.041    0.579    0.563
  BAS ~                                               
    exp       (e2)   -0.062    0.046   -1.341    0.180
  react ~                                             
    exp       (e3)    0.019    0.041    0.459    0.646
  own_proj ~                                          
    BIS     (b1.1)    0.018    0.187    0.094    0.925
    BAS     (b2.1)    0.150    0.086    1.746    0.081
    react   (b3.1)    0.245    0.516    0.475    0.635
    lonel     (l1)   -0.132    0.088   -1.497    0.134
  media ~                                             
    BIS     (b1.2)    0.236    0.330    0.716    0.474
    BAS     (b2.2)    0.221    0.116    1.893    0.058
    react   (b3.2)    0.352    0.505    0.698    0.485
    lonel     (l2)   -0.035    0.133   -0.266    0.790
  security ~                                          
    BIS     (b1.3)    0.371    0.177    2.096    0.036
    BAS     (b2.3)    0.092    0.085    1.085    0.278
    react   (b3.3)   -0.264    0.146   -1.802    0.072
    lonel     (l3)   -0.196    0.118   -1.667    0.096
  sysj ~                                              
    BIS     (b1.4)   -0.016    0.317   -0.049    0.961
    BAS     (b2.4)    0.028    0.205    0.137    0.891
    react   (b3.4)   -0.500    0.465   -1.075    0.282
    lonel     (l4)   -0.571    0.262   -2.182    0.029
  BIS ~                                               
    sex               0.160    0.054    2.981    0.003
  media ~                                             
    sex               0.449    0.097    4.624    0.000
  security ~                                          
    sex               0.192    0.087    2.213    0.027
  BIS ~                                               
    risk              0.015    0.086    0.176    0.860
  own_proj ~                                          
    risk             -0.170    0.100   -1.701    0.089
  sysj ~                                              
    risk             -0.349    0.273   -1.277    0.202
  BAS ~                                               
    edu_dgr           0.079    0.069    1.156    0.248
  react ~                                             
    edu_dgr          -0.025    0.059   -0.415    0.678
  BAS ~                                               
    age               0.005    0.002    2.147    0.032
  react ~                                             
    age              -0.007    0.002   -3.437    0.001
  own_proj ~                                          
    age              -0.010    0.005   -2.094    0.036
    country           0.063    0.064    0.986    0.324
    discr    (cd1)   -0.382    0.948   -0.403    0.687
  media ~                                             
    discr    (cd2)   -0.316    1.109   -0.285    0.776
  security ~                                          
    discr    (cd3)    0.072    0.348    0.207    0.836
  sysj ~                                              
    discr    (cd4)    0.242    0.946    0.256    0.798
  own_proj ~                                          
    exp      (ce1)    0.053    0.053    0.995    0.320
  media ~                                             
    exp      (ce2)    0.027    0.072    0.378    0.706
  security ~                                          
    exp      (ce3)   -0.050    0.043   -1.175    0.240
  sysj ~                                              
    exp      (ce4)    0.269    0.109    2.465    0.014

Covariances:
                             Estimate  Std.Err  z-value  P(>|z|)
 .aut2 ~~                                                       
   .aut1                       -0.294    0.048   -6.139    0.000
 .aut1 ~~                                                       
   .aut3                       -0.100    0.047   -2.121    0.034
   .aut4                       -0.283    0.049   -5.837    0.000
 .aut2 ~~                                                       
   .aut3                        0.143    0.046    3.083    0.002
   .aut4                        0.295    0.046    6.485    0.000
 .aut3 ~~                                                       
   .aut4                        0.078    0.045    1.748    0.080
 .uncer1 ~~                                                     
   .uncer2                      0.096    0.043    2.216    0.027
   .uncer3                      0.020    0.046    0.441    0.659
   .uncer4                      0.078    0.047    1.636    0.102
 .uncer2 ~~                                                     
   .uncer3                      0.059    0.056    1.063    0.288
   .uncer4                     -0.039    0.046   -0.846    0.397
 .uncer3 ~~                                                     
   .uncer4                      0.016    0.048    0.339    0.735
 .agenc1 ~~                                                     
   .agenc2                     -0.001    0.047   -0.025    0.980
   .agenc3                     -0.036    0.035   -1.019    0.308
   .agenc4                     -0.063    0.032   -1.944    0.052
 .agenc2 ~~                                                     
   .agenc3                     -0.030    0.039   -0.765    0.445
   .agenc4                     -0.094    0.041   -2.262    0.024
 .agenc3 ~~                                                     
   .agenc4                      0.090    0.031    2.942    0.003
 .reac2 ~~                                                      
   .reac3                       0.065    0.044    1.475    0.140
 .reac5 ~~                                                      
   .reac6                       0.213    0.042    5.013    0.000
 .reac4 ~~                                                      
   .host6                       0.310    0.055    5.647    0.000
 .reac1 ~~                                                      
   .host6                       0.090    0.027    3.375    0.001
 .reac6 ~~                                                      
   .host5                       0.078    0.026    2.970    0.003
 .reac4 ~~                                                      
   .host3                       0.140    0.037    3.771    0.000
   .host5                       0.118    0.035    3.353    0.001
 .host1 ~~                                                      
   .host5                       0.150    0.046    3.293    0.001
   .host3                       0.063    0.030    2.091    0.037
 .host2 ~~                                                      
   .host3                       0.092    0.026    3.511    0.000
   .host5                       0.085    0.025    3.421    0.001
 .host1 ~~                                                      
   .host2                       0.052    0.027    1.966    0.049
 .host2 ~~                                                      
   .host6                       0.115    0.028    4.035    0.000
 .host3 ~~                                                      
   .host5                       0.133    0.041    3.270    0.001
   .host6                       0.179    0.043    4.150    0.000
 .host5 ~~                                                      
   .host6                       0.146    0.040    3.687    0.000
 .fear6 ~~                                                      
   .bis2                        0.145    0.027    5.261    0.000
 .fear2 ~~                                                      
   .bis2                        0.102    0.026    3.897    0.000
   .fear6                       0.084    0.025    3.303    0.001
 .reac4 ~~                                                      
   .host2                       0.079    0.027    2.908    0.004
 .bis3 ~~                                                       
   .bis4                        0.065    0.033    1.969    0.049
 .bash1 ~~                                                      
   .bash2                       0.156    0.039    4.049    0.000
 .bash4 ~~                                                      
   .bash5                       0.129    0.051    2.516    0.012
 .SJ2 ~~                                                        
   .SJ5                         0.591    0.118    5.010    0.000
   .SJ4                         0.626    0.129    4.868    0.000
 .SJ4 ~~                                                        
   .SJ5                         0.421    0.108    3.899    0.000
 .SJ5 ~~                                                        
   .SJ6                         0.462    0.130    3.550    0.000
 .SJ2 ~~                                                        
   .SJ6                         0.523    0.148    3.534    0.000
 .SJ7 ~~                                                        
   .SJ8                         0.366    0.105    3.488    0.000
 .SJ6 ~~                                                        
   .SJ7                         0.379    0.113    3.350    0.001
 .SJ3 ~~                                                        
   .SJ6                         0.314    0.121    2.587    0.010
 .SJ6 ~~                                                        
   .SJ8                         0.289    0.126    2.289    0.022
 .SJ4 ~~                                                        
   .SJ6                         0.218    0.146    1.496    0.135
   .SJ8                         0.177    0.099    1.778    0.075
 .lrom1 ~~                                                      
   .lrom2                       5.041    0.327   15.439    0.000
   .lrom3                      -2.483    0.315   -7.888    0.000
   .lrom4                       5.093    0.310   16.422    0.000
   .lrom5                      -3.017    0.311   -9.712    0.000
 .lrom2 ~~                                                      
   .lrom3                      -2.390    0.320   -7.463    0.000
   .lrom4                       4.952    0.319   15.531    0.000
   .lrom5                      -2.951    0.320   -9.233    0.000
 .lrom3 ~~                                                      
   .lrom4                      -2.304    0.303   -7.601    0.000
   .lrom5                       2.537    0.328    7.741    0.000
 .lrom4 ~~                                                      
   .lrom5                      -2.992    0.301   -9.942    0.000
 .lsoc1 ~~                                                      
   .lsoc2                       0.297    0.166    1.791    0.073
   .lsoc3                      -0.311    0.165   -1.885    0.059
   .lsoc4                       0.436    0.160    2.727    0.006
 .lsoc5 ~~                                                      
   .lsoc1                      -0.420    0.174   -2.419    0.016
 .lsoc2 ~~                                                      
   .lsoc3                      -0.286    0.147   -1.949    0.051
   .lsoc4                       0.345    0.129    2.668    0.008
 .lsoc5 ~~                                                      
   .lsoc2                      -0.302    0.152   -1.981    0.048
 .lsoc4 ~~                                                      
   .lsoc3                      -0.310    0.138   -2.249    0.025
 .lsoc5 ~~                                                      
   .lsoc3                       0.503    0.162    3.100    0.002
   .lsoc4                      -0.328    0.139   -2.358    0.018
 .lfam1 ~~                                                      
   .lfam2                       0.166    0.142    1.170    0.242
   .lfam3                      -0.565    0.183   -3.079    0.002
   .lfam4                      -0.372    0.172   -2.158    0.031
   .lfam5                      -0.343    0.166   -2.058    0.040
 .lfam2 ~~                                                      
   .lfam3                      -0.286    0.127   -2.255    0.024
   .lfam4                      -0.321    0.123   -2.610    0.009
   .lfam5                      -0.396    0.127   -3.111    0.002
 .lfam3 ~~                                                      
   .lfam4                       0.702    0.164    4.283    0.000
   .lfam5                       0.629    0.158    3.974    0.000
 .lfam4 ~~                                                      
   .lfam5                       0.493    0.155    3.192    0.001
 .reac1 ~~                                                      
   .reac7                       0.132    0.041    3.202    0.001
 .creative ~~                                                   
   .social_media                0.050    0.031    1.620    0.105
 .own_projects ~~                                               
   .creative                    0.141    0.070    2.003    0.045
 .social_media ~~                                               
   .information                 0.389    0.042    9.209    0.000
 .digital_social_contacts ~~                                    
   .washing_hands               0.126    0.047    2.689    0.007
 .doing_sports ~~                                               
   .dgtl_scl_cntct              0.224    1.465    0.153    0.879
 .own_projects ~~                                               
   .dgtl_scl_cntct              0.188    1.193    0.158    0.875
 .creative ~~                                                   
   .dgtl_scl_cntct              0.169    1.233    0.137    0.891
 .washing_hands ~~                                              
   .stockpiling                -0.054    0.058   -0.924    0.355
 .disregard_curfew ~~                                           
   .stockpiling                 0.096    0.053    1.813    0.070
 .BIS ~~                                                        
   .react                       0.041    0.022    1.917    0.055
  lonel ~~                                                      
    discr                       0.083    0.029    2.846    0.004
    exp                         0.024    0.043    0.553    0.580
  discr ~~                                                      
    exp                         0.026    0.027    0.965    0.335
 .own_proj ~~                                                   
   .media                      -0.033    1.194   -0.028    0.978
   .security                    0.032    0.020    1.609    0.108
   .sysj                        0.027    0.051    0.520    0.603
 .media ~~                                                      
   .security                    0.041    0.038    1.094    0.274
   .sysj                        0.156    0.065    2.415    0.016
 .security ~~                                                   
   .sysj                       -0.028    0.039   -0.714    0.475

Variances:
                   Estimate  Std.Err  z-value  P(>|z|)
   .own_projects      0.310    0.078    3.948    0.000
   .doing_sports      0.496    0.139    3.561    0.000
   .creative          0.534    0.088    6.061    0.000
   .dgtl_scl_cntct    0.519    5.808    0.089    0.929
   .social_media      0.685    0.060   11.509    0.000
   .washing_hands     0.643    0.088    7.294    0.000
   .disregard_crfw    0.182    0.102    1.779    0.075
   .stockpiling       0.434    0.067    6.471    0.000
   .information       0.790    0.048   16.336    0.000
   .SJ1               0.825    0.148    5.564    0.000
   .SJ2               1.872    0.162   11.583    0.000
   .SJ3               2.300    0.191   12.027    0.000
   .SJ4               1.908    0.151   12.623    0.000
   .SJ5               1.471    0.149    9.902    0.000
   .SJ6               2.330    0.195   11.947    0.000
   .SJ7               1.445    0.144   10.021    0.000
   .SJ8               1.815    0.173   10.505    0.000
   .lsoc5             0.937    0.199    4.710    0.000
   .lsoc1             1.363    0.216    6.308    0.000
   .lsoc2             0.836    0.152    5.500    0.000
   .lsoc4             0.851    0.139    6.136    0.000
   .lsoc3             0.871    0.182    4.793    0.000
   .lfam1             1.375    0.256    5.375    0.000
   .lfam2             0.781    0.167    4.675    0.000
   .lfam3             1.193    0.180    6.642    0.000
   .lfam4             1.026    0.184    5.571    0.000
   .lfam5             1.353    0.203    6.680    0.000
   .lrom1             5.545    0.319   17.388    0.000
   .lrom2             5.461    0.334   16.370    0.000
   .lrom3             3.730    0.359   10.387    0.000
   .lrom4             5.370    0.299   17.966    0.000
   .lrom5             3.921    0.347   11.284    0.000
   .aut2              0.749    0.053   14.073    0.000
   .aut1              0.700    0.061   11.563    0.000
   .aut3              0.824    0.060   13.794    0.000
   .aut4              0.665    0.058   11.423    0.000
   .uncer1            0.661    0.061   10.890    0.000
   .uncer2            1.039    0.070   14.786    0.000
   .uncer3            0.983    0.072   13.646    0.000
   .uncer4            0.623    0.069    8.991    0.000
   .agenc1            0.584    0.051   11.456    0.000
   .agenc2            1.012    0.068   14.893    0.000
   .agenc3            0.423    0.042   10.086    0.000
   .agenc4            0.471    0.040   11.800    0.000
   .expec1            0.583    0.074    7.836    0.000
   .expec2            0.617    0.069    8.881    0.000
   .expec3            0.479    0.060    7.963    0.000
   .fear2             0.385    0.038   10.054    0.000
   .fear5             0.386    0.038   10.059    0.000
   .fear6             0.317    0.032    9.992    0.000
   .bis1              0.903    0.068   13.373    0.000
   .bis2              0.315    0.032    9.855    0.000
   .bis3              0.335    0.036    9.382    0.000
   .bis4              0.375    0.039    9.518    0.000
   .bis5              0.420    0.046    9.230    0.000
   .reac2             0.703    0.066   10.655    0.000
   .reac1             0.641    0.053   12.082    0.000
   .reac3             0.509    0.055    9.267    0.000
   .reac4             0.769    0.066   11.709    0.000
   .reac5             0.589    0.071    8.341    0.000
   .reac6             0.539    0.068    7.887    0.000
   .reac7             0.806    0.065   12.486    0.000
   .host1             0.381    0.060    6.367    0.000
   .host2             0.265    0.034    7.675    0.000
   .host3             0.279    0.053    5.296    0.000
   .host4             0.588    0.052   11.210    0.000
   .host5             0.415    0.057    7.315    0.000
   .host6             0.551    0.061    9.041    0.000
   .bash1             0.447    0.046    9.641    0.000
   .bash2             0.388    0.044    8.776    0.000
   .bash3             0.463    0.045   10.235    0.000
   .bash4             0.484    0.062    7.837    0.000
   .bash5             0.457    0.057    8.013    0.000
   .own_proj          0.184    0.073    2.504    0.012
   .media             0.122    5.805    0.021    0.983
   .security          0.110    0.066    1.670    0.095
   .sysj              1.372    0.179    7.657    0.000
    lonel             0.419    0.193    2.167    0.030
    discr             0.163    0.041    3.980    0.000
    exp               0.696    0.097    7.144    0.000
   .BIS               0.151    0.030    5.093    0.000
   .react             0.116    0.032    3.677    0.000
   .BAS               0.250    0.041    6.153    0.000

Defined Parameters:
                   Estimate  Std.Err  z-value  P(>|z|)
    ab1.1             0.024    0.276    0.088    0.930
    ab1.2             0.327    0.490    0.668    0.504
    ab1.3             0.514    0.260    1.975    0.048
    ab1.4            -0.022    0.452   -0.048    0.962
    ab2.1            -0.186    0.111   -1.675    0.094
    ab2.2            -0.273    0.152   -1.797    0.072
    ab2.3            -0.114    0.107   -1.061    0.289
    ab2.4            -0.035    0.264   -0.132    0.895
    ab3.1             0.296    0.763    0.387    0.699
    ab3.2             0.425    0.727    0.585    0.559
    ab3.3            -0.318    0.188   -1.690    0.091
    ab3.4            -0.603    0.602   -1.003    0.316
    eb1.1             0.000    0.009    0.044    0.965
    eb1.2             0.006    0.021    0.268    0.789
    eb1.3             0.009    0.018    0.484    0.628
    eb1.4            -0.000    0.014   -0.026    0.979
    eb2.1            -0.009    0.010   -0.927    0.354
    eb2.2            -0.014    0.014   -1.004    0.315
    eb2.3            -0.006    0.008   -0.691    0.490
    eb2.4            -0.002    0.016   -0.106    0.916
    eb3.1             0.005    0.031    0.145    0.884
    eb3.2             0.007    0.032    0.202    0.840
    eb3.3            -0.005    0.010   -0.495    0.621
    eb3.4            -0.009    0.028   -0.338    0.736
    tot.d1.1         -0.358    0.806   -0.444    0.657
    tot.d1.2          0.011    0.787    0.014    0.989
    tot.d1.3          0.586    0.261    2.244    0.025
    tot.d2.1         -0.568    0.931   -0.610    0.542
    tot.d2.2         -0.589    1.085   -0.543    0.587
    tot.d2.3         -0.042    0.324   -0.130    0.897
    tot.d3.1         -0.086    0.353   -0.245    0.807
    tot.d3.2          0.109    0.586    0.186    0.853
    tot.d3.3         -0.246    0.269   -0.914    0.361
```

```
sem.cov.fit <- sem(sem.cov.mod, data = df)

sem.cov.sum <- summary(sem.cov.fit, fit.measures = T)
```

```
lavaan 0.6-7 ended normally after 193 iterations

  Estimator                                         ML
  Optimization method                           NLMINB
  Number of free parameters                        290
                                                      
  Number of observations                           395
                                                      
Model Test User Model:
                                                      
  Test statistic                              4642.077
  Degrees of freedom                              2776
  P-value (Chi-square)                           0.000

Model Test Baseline Model:

  Test statistic                             17239.179
  Degrees of freedom                              2993
  P-value                                        0.000

User Model versus Baseline Model:

  Comparative Fit Index (CFI)                    0.869
  Tucker-Lewis Index (TLI)                       0.859

Loglikelihood and Information Criteria:

  Loglikelihood user model (H0)             -36911.405
  Loglikelihood unrestricted model (H1)     -34590.366
                                                      
  Akaike (AIC)                               74402.809
  Bayesian (BIC)                             75556.686
  Sample-size adjusted Bayesian (BIC)        74636.515

Root Mean Square Error of Approximation:

  RMSEA                                          0.041
  90 Percent confidence interval - lower         0.039
  90 Percent confidence interval - upper         0.043
  P-value RMSEA <= 0.05                          1.000

Standardized Root Mean Square Residual:

  SRMR                                           0.067

Parameter Estimates:

  Standard errors                             Standard
  Information                                 Expected
  Information saturated (h1) model          Structured

Latent Variables:
                   Estimate  Std.Err  z-value  P(>|z|)
  own_proj =~                                         
    own_projects      1.000                           
    doing_sports      1.353    0.190    7.110    0.000
    creative          1.005    0.098   10.253    0.000
  media =~                                            
    dgtl_scl_cntct    1.000                           
    social_media      0.407    0.120    3.388    0.001
  security =~                                         
    washing_hands     1.000                           
    disregard_crfw   -0.918    0.200   -4.582    0.000
    stockpiling       0.572    0.196    2.924    0.003
    information       0.213    0.126    1.688    0.091
  sysj =~                                             
    SJ1               1.000                           
    SJ2               0.506    0.066    7.665    0.000
    SJ3              -0.789    0.080   -9.886    0.000
    SJ4               0.404    0.065    6.166    0.000
    SJ5               0.226    0.055    4.149    0.000
    SJ6               0.545    0.081    6.708    0.000
    SJ7              -0.789    0.074  -10.676    0.000
    SJ8               0.604    0.072    8.361    0.000
  lonel =~                                            
    lsoc5             1.000                           
    lsoc1            -1.029    0.123   -8.352    0.000
    lsoc2            -0.906    0.104   -8.726    0.000
    lsoc4            -0.864    0.100   -8.624    0.000
    lsoc3             0.960    0.089   10.847    0.000
    lfam1             1.231    0.199    6.193    0.000
    lfam2             0.960    0.153    6.268    0.000
    lfam3            -1.136    0.184   -6.170    0.000
    lfam4            -1.019    0.167   -6.093    0.000
    lfam5            -0.971    0.173   -5.620    0.000
    lrom1            -1.143    0.258   -4.435    0.000
    lrom2            -1.151    0.257   -4.484    0.000
    lrom3             1.421    0.243    5.856    0.000
    lrom4            -1.072    0.251   -4.276    0.000
    lrom5             1.276    0.236    5.405    0.000
  discr =~                                            
    aut2              1.000                           
    aut1             -1.222    0.139   -8.764    0.000
    aut3              1.259    0.171    7.363    0.000
    aut4              0.790    0.109    7.280    0.000
    uncer1           -1.527    0.209   -7.323    0.000
    uncer2            1.108    0.189    5.871    0.000
    uncer3            0.895    0.170    5.272    0.000
    uncer4            2.033    0.259    7.855    0.000
    agenc1            1.376    0.189    7.262    0.000
    agenc2            1.239    0.197    6.290    0.000
    agenc3           -1.401    0.184   -7.607    0.000
    agenc4           -1.211    0.168   -7.220    0.000
  exp =~                                              
    expec1            1.000                           
    expec2           -1.055    0.090  -11.674    0.000
    expec3           -0.921    0.079  -11.665    0.000
  BIS =~                                              
    fear2             1.000                           
    fear5             1.263    0.078   16.223    0.000
    fear6             1.147    0.061   18.701    0.000
    bis1              0.771    0.082    9.451    0.000
    bis2              1.156    0.060   19.415    0.000
    bis3              1.180    0.073   16.147    0.000
    bis4              1.250    0.077   16.151    0.000
    bis5              1.113    0.073   15.154    0.000
  react =~                                            
    reac2             1.000                           
    reac1            -0.940    0.103   -9.158    0.000
    reac3             1.337    0.114   11.735    0.000
    reac4             0.864    0.103    8.406    0.000
    reac5             0.501    0.078    6.401    0.000
    reac6             0.562    0.078    7.180    0.000
    reac7             1.140    0.120    9.528    0.000
    host1             0.308    0.060    5.155    0.000
    host2             0.292    0.051    5.703    0.000
    host3             0.320    0.053    6.013    0.000
    host4             1.025    0.104    9.821    0.000
    host5             0.409    0.066    6.236    0.000
    host6             0.883    0.095    9.254    0.000
  BAS =~                                              
    bash1             1.000                           
    bash2             1.033    0.059   17.653    0.000
    bash3             0.854    0.072   11.825    0.000
    bash4             1.013    0.082   12.303    0.000
    bash5             0.997    0.081   12.372    0.000

Regressions:
                   Estimate  Std.Err  z-value  P(>|z|)
  BIS ~                                               
    discr     (a1)    1.384    0.183    7.578    0.000
  BAS ~                                               
    discr     (a2)   -1.238    0.175   -7.082    0.000
  react ~                                             
    discr     (a3)    1.206    0.175    6.888    0.000
  BIS ~                                               
    exp       (e1)    0.024    0.035    0.669    0.503
  BAS ~                                               
    exp       (e2)   -0.062    0.045   -1.382    0.167
  react ~                                             
    exp       (e3)    0.019    0.034    0.551    0.582
  own_proj ~                                          
    BIS     (b1.1)    0.018    0.097    0.181    0.856
    BAS     (b2.1)    0.150    0.075    2.009    0.044
    react   (b3.1)    0.245    0.127    1.924    0.054
    lonel     (l1)   -0.132    0.061   -2.166    0.030
  media ~                                             
    BIS     (b1.2)    0.236    0.133    1.774    0.076
    BAS     (b2.2)    0.221    0.096    2.295    0.022
    react   (b3.2)    0.352    0.152    2.315    0.021
    lonel     (l2)   -0.035    0.078   -0.453    0.651
  security ~                                          
    BIS     (b1.3)    0.371    0.107    3.458    0.001
    BAS     (b2.3)    0.092    0.062    1.475    0.140
    react   (b3.3)   -0.264    0.107   -2.467    0.014
    lonel     (l3)   -0.196    0.063   -3.097    0.002
  sysj ~                                              
    BIS     (b1.4)   -0.016    0.229   -0.068    0.945
    BAS     (b2.4)    0.028    0.170    0.165    0.869
    react   (b3.4)   -0.500    0.271   -1.845    0.065
    lonel     (l4)   -0.571    0.154   -3.709    0.000
  BIS ~                                               
    sex               0.160    0.051    3.166    0.002
  media ~                                             
    sex               0.449    0.081    5.565    0.000
  security ~                                          
    sex               0.192    0.062    3.084    0.002
  BIS ~                                               
    risk              0.015    0.070    0.216    0.829
  own_proj ~                                          
    risk             -0.170    0.087   -1.965    0.049
  sysj ~                                              
    risk             -0.349    0.203   -1.719    0.086
  BAS ~                                               
    edu_dgr           0.079    0.066    1.196    0.232
  react ~                                             
    edu_dgr          -0.025    0.046   -0.529    0.597
  BAS ~                                               
    age               0.005    0.002    2.368    0.018
  react ~                                             
    age              -0.007    0.002   -4.491    0.000
  own_proj ~                                          
    age              -0.010    0.002   -4.076    0.000
    country           0.063    0.061    1.044    0.297
    discr    (cd1)   -0.382    0.258   -1.482    0.138
  media ~                                             
    discr    (cd2)   -0.316    0.320   -0.988    0.323
  security ~                                          
    discr    (cd3)    0.072    0.202    0.356    0.722
  sysj ~                                              
    discr    (cd4)    0.242    0.560    0.433    0.665
  own_proj ~                                          
    exp      (ce1)    0.053    0.040    1.338    0.181
  media ~                                             
    exp      (ce2)    0.027    0.052    0.523    0.601
  security ~                                          
    exp      (ce3)   -0.050    0.035   -1.447    0.148
  sysj ~                                              
    exp      (ce4)    0.269    0.096    2.811    0.005

Covariances:
                             Estimate  Std.Err  z-value  P(>|z|)
 .aut2 ~~                                                       
   .aut1                       -0.294    0.041   -7.139    0.000
 .aut1 ~~                                                       
   .aut3                       -0.100    0.041   -2.471    0.013
   .aut4                       -0.283    0.039   -7.312    0.000
 .aut2 ~~                                                       
   .aut3                        0.143    0.042    3.402    0.001
   .aut4                        0.295    0.040    7.437    0.000
 .aut3 ~~                                                       
   .aut4                        0.078    0.039    2.012    0.044
 .uncer1 ~~                                                     
   .uncer2                      0.096    0.045    2.141    0.032
   .uncer3                      0.020    0.043    0.465    0.642
   .uncer4                      0.078    0.038    2.067    0.039
 .uncer2 ~~                                                     
   .uncer3                      0.059    0.053    1.127    0.260
   .uncer4                     -0.039    0.045   -0.858    0.391
 .uncer3 ~~                                                     
   .uncer4                      0.016    0.044    0.372    0.710
 .agenc1 ~~                                                     
   .agenc2                     -0.001    0.041   -0.028    0.978
   .agenc3                     -0.036    0.028   -1.284    0.199
   .agenc4                     -0.063    0.029   -2.154    0.031
 .agenc2 ~~                                                     
   .agenc3                     -0.030    0.036   -0.837    0.402
   .agenc4                     -0.094    0.038   -2.499    0.012
 .agenc3 ~~                                                     
   .agenc4                      0.090    0.026    3.482    0.000
 .reac2 ~~                                                      
   .reac3                       0.065    0.036    1.790    0.073
 .reac5 ~~                                                      
   .reac6                       0.213    0.031    6.904    0.000
 .reac4 ~~                                                      
   .host6                       0.310    0.040    7.782    0.000
 .reac1 ~~                                                      
   .host6                       0.090    0.026    3.526    0.000
 .reac6 ~~                                                      
   .host5                       0.078    0.019    4.044    0.000
 .reac4 ~~                                                      
   .host3                       0.140    0.025    5.607    0.000
   .host5                       0.118    0.027    4.304    0.000
 .host1 ~~                                                      
   .host5                       0.150    0.020    7.408    0.000
   .host3                       0.063    0.015    4.186    0.000
 .host2 ~~                                                      
   .host3                       0.092    0.015    6.282    0.000
   .host5                       0.085    0.017    4.969    0.000
 .host1 ~~                                                      
   .host2                       0.052    0.016    3.347    0.001
 .host2 ~~                                                      
   .host6                       0.115    0.021    5.454    0.000
 .host3 ~~                                                      
   .host5                       0.133    0.018    7.401    0.000
   .host6                       0.179    0.023    7.871    0.000
 .host5 ~~                                                      
   .host6                       0.146    0.024    5.976    0.000
 .fear6 ~~                                                      
   .bis2                        0.145    0.022    6.455    0.000
 .fear2 ~~                                                      
   .bis2                        0.102    0.022    4.545    0.000
   .fear6                       0.084    0.022    3.809    0.000
 .reac4 ~~                                                      
   .host2                       0.079    0.024    3.339    0.001
 .bis3 ~~                                                       
   .bis4                        0.065    0.023    2.781    0.005
 .bash1 ~~                                                      
   .bash2                       0.156    0.034    4.604    0.000
 .bash4 ~~                                                      
   .bash5                       0.129    0.035    3.672    0.000
 .SJ2 ~~                                                        
   .SJ5                         0.591    0.092    6.406    0.000
   .SJ4                         0.626    0.105    5.971    0.000
 .SJ4 ~~                                                        
   .SJ5                         0.421    0.089    4.759    0.000
 .SJ5 ~~                                                        
   .SJ6                         0.462    0.100    4.615    0.000
 .SJ2 ~~                                                        
   .SJ6                         0.523    0.119    4.408    0.000
 .SJ7 ~~                                                        
   .SJ8                         0.366    0.099    3.697    0.000
 .SJ6 ~~                                                        
   .SJ7                         0.379    0.108    3.524    0.000
 .SJ3 ~~                                                        
   .SJ6                         0.314    0.124    2.526    0.012
 .SJ6 ~~                                                        
   .SJ8                         0.289    0.113    2.560    0.010
 .SJ4 ~~                                                        
   .SJ6                         0.218    0.116    1.878    0.060
   .SJ8                         0.177    0.095    1.868    0.062
 .lrom1 ~~                                                      
   .lrom2                       5.041    0.395   12.752    0.000
   .lrom3                      -2.483    0.286   -8.687    0.000
   .lrom4                       5.093    0.394   12.926    0.000
   .lrom5                      -3.017    0.302  -10.001    0.000
 .lrom2 ~~                                                      
   .lrom3                      -2.390    0.282   -8.472    0.000
   .lrom4                       4.952    0.388   12.770    0.000
   .lrom5                      -2.951    0.298   -9.887    0.000
 .lrom3 ~~                                                      
   .lrom4                      -2.304    0.277   -8.318    0.000
   .lrom5                       2.537    0.258    9.815    0.000
 .lrom4 ~~                                                      
   .lrom5                      -2.992    0.297  -10.087    0.000
 .lsoc1 ~~                                                      
   .lsoc2                       0.297    0.077    3.886    0.000
   .lsoc3                      -0.311    0.079   -3.920    0.000
   .lsoc4                       0.436    0.079    5.542    0.000
 .lsoc5 ~~                                                      
   .lsoc1                      -0.420    0.084   -4.987    0.000
 .lsoc2 ~~                                                      
   .lsoc3                      -0.286    0.065   -4.380    0.000
   .lsoc4                       0.345    0.064    5.403    0.000
 .lsoc5 ~~                                                      
   .lsoc2                      -0.302    0.068   -4.446    0.000
 .lsoc4 ~~                                                      
   .lsoc3                      -0.310    0.065   -4.763    0.000
 .lsoc5 ~~                                                      
   .lsoc3                       0.503    0.075    6.713    0.000
   .lsoc4                      -0.328    0.068   -4.833    0.000
 .lfam1 ~~                                                      
   .lfam2                       0.166    0.081    2.051    0.040
   .lfam3                      -0.565    0.106   -5.353    0.000
   .lfam4                      -0.372    0.093   -3.985    0.000
   .lfam5                      -0.343    0.099   -3.454    0.001
 .lfam2 ~~                                                      
   .lfam3                      -0.286    0.077   -3.699    0.000
   .lfam4                      -0.321    0.072   -4.446    0.000
   .lfam5                      -0.396    0.078   -5.049    0.000
 .lfam3 ~~                                                      
   .lfam4                       0.702    0.095    7.362    0.000
   .lfam5                       0.629    0.099    6.369    0.000
 .lfam4 ~~                                                      
   .lfam5                       0.493    0.089    5.575    0.000
 .reac1 ~~                                                      
   .reac7                       0.132    0.040    3.309    0.001
 .creative ~~                                                   
   .social_media                0.050    0.026    1.948    0.051
 .own_projects ~~                                               
   .creative                    0.141    0.040    3.497    0.000
 .social_media ~~                                               
   .information                 0.389    0.042    9.214    0.000
 .digital_social_contacts ~~                                    
   .washing_hands               0.126    0.035    3.611    0.000
 .doing_sports ~~                                               
   .dgtl_scl_cntct              0.224    0.078    2.878    0.004
 .own_projects ~~                                               
   .dgtl_scl_cntct              0.188    0.058    3.234    0.001
 .creative ~~                                                   
   .dgtl_scl_cntct              0.169    0.061    2.769    0.006
 .washing_hands ~~                                              
   .stockpiling                -0.054    0.039   -1.373    0.170
 .disregard_curfew ~~                                           
   .stockpiling                 0.096    0.031    3.110    0.002
 .BIS ~~                                                        
   .react                       0.041    0.014    2.966    0.003
  lonel ~~                                                      
    discr                       0.083    0.022    3.853    0.000
    exp                         0.024    0.038    0.628    0.530
  discr ~~                                                      
    exp                         0.026    0.021    1.223    0.221
 .own_proj ~~                                                   
   .media                      -0.033    0.053   -0.630    0.529
   .security                    0.032    0.015    2.092    0.036
   .sysj                        0.027    0.039    0.676    0.499
 .media ~~                                                      
   .security                    0.041    0.021    1.951    0.051
   .sysj                        0.156    0.053    2.945    0.003
 .security ~~                                                   
   .sysj                       -0.028    0.034   -0.833    0.405

Variances:
                   Estimate  Std.Err  z-value  P(>|z|)
   .own_projects      0.310    0.041    7.554    0.000
   .doing_sports      0.496    0.070    7.078    0.000
   .creative          0.534    0.056    9.574    0.000
   .dgtl_scl_cntct    0.519    0.085    6.138    0.000
   .social_media      0.685    0.051   13.538    0.000
   .washing_hands     0.643    0.058   11.068    0.000
   .disregard_crfw    0.182    0.033    5.586    0.000
   .stockpiling       0.434    0.042   10.314    0.000
   .information       0.790    0.056   13.989    0.000
   .SJ1               0.825    0.122    6.776    0.000
   .SJ2               1.872    0.141   13.280    0.000
   .SJ3               2.300    0.184   12.491    0.000
   .SJ4               1.908    0.141   13.536    0.000
   .SJ5               1.471    0.106   13.852    0.000
   .SJ6               2.330    0.182   12.777    0.000
   .SJ7               1.445    0.133   10.884    0.000
   .SJ8               1.815    0.146   12.454    0.000
   .lsoc5             0.937    0.091   10.347    0.000
   .lsoc1             1.363    0.121   11.217    0.000
   .lsoc2             0.836    0.079   10.592    0.000
   .lsoc4             0.851    0.078   10.906    0.000
   .lsoc3             0.871    0.084   10.372    0.000
   .lfam1             1.375    0.137   10.069    0.000
   .lfam2             0.781    0.079    9.857    0.000
   .lfam3             1.193    0.118   10.131    0.000
   .lfam4             1.026    0.099   10.331    0.000
   .lfam5             1.353    0.120   11.325    0.000
   .lrom1             5.545    0.414   13.386    0.000
   .lrom2             5.461    0.409   13.367    0.000
   .lrom3             3.730    0.296   12.583    0.000
   .lrom4             5.370    0.399   13.445    0.000
   .lrom5             3.921    0.304   12.905    0.000
   .aut2              0.749    0.055   13.632    0.000
   .aut1              0.700    0.052   13.390    0.000
   .aut3              0.824    0.061   13.454    0.000
   .aut4              0.665    0.048   13.755    0.000
   .uncer1            0.661    0.052   12.660    0.000
   .uncer2            1.039    0.077   13.563    0.000
   .uncer3            0.983    0.072   13.712    0.000
   .uncer4            0.623    0.054   11.607    0.000
   .agenc1            0.584    0.045   12.916    0.000
   .agenc2            1.012    0.075   13.502    0.000
   .agenc3            0.423    0.034   12.468    0.000
   .agenc4            0.471    0.036   12.957    0.000
   .expec1            0.583    0.064    9.140    0.000
   .expec2            0.617    0.070    8.868    0.000
   .expec3            0.479    0.053    8.977    0.000
   .fear2             0.385    0.031   12.339    0.000
   .fear5             0.386    0.033   11.735    0.000
   .fear6             0.317    0.028   11.474    0.000
   .bis1              0.903    0.066   13.692    0.000
   .bis2              0.315    0.028   11.424    0.000
   .bis3              0.335    0.029   11.424    0.000
   .bis4              0.375    0.033   11.421    0.000
   .bis5              0.420    0.034   12.410    0.000
   .reac2             0.703    0.055   12.790    0.000
   .reac1             0.641    0.050   12.808    0.000
   .reac3             0.509    0.045   11.246    0.000
   .reac4             0.769    0.058   13.288    0.000
   .reac5             0.589    0.043   13.735    0.000
   .reac6             0.539    0.039   13.698    0.000
   .reac7             0.806    0.064   12.664    0.000
   .host1             0.381    0.028   13.871    0.000
   .host2             0.265    0.019   13.836    0.000
   .host3             0.279    0.020   13.891    0.000
   .host4             0.588    0.046   12.725    0.000
   .host5             0.415    0.029   14.124    0.000
   .host6             0.551    0.043   12.823    0.000
   .bash1             0.447    0.043   10.316    0.000
   .bash2             0.388    0.040    9.685    0.000
   .bash3             0.463    0.039   11.838    0.000
   .bash4             0.484    0.046   10.555    0.000
   .bash5             0.457    0.044   10.483    0.000
   .own_proj          0.184    0.038    4.903    0.000
   .media             0.122    0.078    1.569    0.117
   .security          0.110    0.035    3.144    0.002
   .sysj              1.372    0.174    7.895    0.000
    lonel             0.419    0.093    4.515    0.000
    discr             0.163    0.039    4.145    0.000
    exp               0.696    0.094    7.374    0.000
   .BIS               0.151    0.023    6.533    0.000
   .react             0.116    0.024    4.819    0.000
   .BAS               0.250    0.038    6.539    0.000

Defined Parameters:
                   Estimate  Std.Err  z-value  P(>|z|)
    ab1.1             0.024    0.134    0.181    0.856
    ab1.2             0.327    0.189    1.731    0.083
    ab1.3             0.514    0.160    3.209    0.001
    ab1.4            -0.022    0.317   -0.068    0.945
    ab2.1            -0.186    0.095   -1.958    0.050
    ab2.2            -0.273    0.124   -2.210    0.027
    ab2.3            -0.114    0.079   -1.449    0.147
    ab2.4            -0.035    0.210   -0.165    0.869
    ab3.1             0.296    0.158    1.874    0.061
    ab3.2             0.425    0.190    2.239    0.025
    ab3.3            -0.318    0.133   -2.388    0.017
    ab3.4            -0.603    0.333   -1.811    0.070
    eb1.1             0.000    0.002    0.176    0.860
    eb1.2             0.006    0.009    0.629    0.529
    eb1.3             0.009    0.013    0.656    0.512
    eb1.4            -0.000    0.005   -0.068    0.946
    eb2.1            -0.009    0.008   -1.131    0.258
    eb2.2            -0.014    0.011   -1.183    0.237
    eb2.3            -0.006    0.006   -1.021    0.307
    eb2.4            -0.002    0.011   -0.163    0.870
    eb3.1             0.005    0.009    0.533    0.594
    eb3.2             0.007    0.012    0.538    0.591
    eb3.3            -0.005    0.009   -0.540    0.589
    eb3.4            -0.009    0.018   -0.524    0.600
    tot.d1.1         -0.358    0.223   -1.603    0.109
    tot.d1.2          0.011    0.261    0.043    0.966
    tot.d1.3          0.586    0.206    2.848    0.004
    tot.d2.1         -0.568    0.230   -2.465    0.014
    tot.d2.2         -0.589    0.286   -2.060    0.039
    tot.d2.3         -0.042    0.176   -0.239    0.811
    tot.d3.1         -0.086    0.207   -0.418    0.676
    tot.d3.2          0.109    0.278    0.390    0.696
    tot.d3.3         -0.246    0.185   -1.329    0.184
```

```
parameterestimates(sem.cov.fit)%>%
filter(op%in%c("~"))%>%
filter(!str_detect(lhs,"tot."))%>%
mutate_at(c("est","se","z","ci.lower","ci.upper"),round,2)%>%
mutate(Regression=paste(rhs,"-->",lhs),
CI95=paste0("[",ci.lower,",",ci.upper,"]"))%>%
select(Regression,b=est,"SE"=se,"z-value"=z,"p-value"=pvalue,"95%CI"=CI95)%>%
kable(caption="Regressionweightsand95%confidenceintervals",
digits=3)%>%
kable_styling(bootstrap_options="striped")%>%
write_file("cov_regressiontable.html")
```

```
parameterestimates(sem.cov.fit)%>%
filter(op%in%c(":="))%>%
filter(!str_detect(lhs,"tot."))%>%
mutate_at(c("est","se","z","ci.lower","ci.upper"),round,2)%>%
mutate(CI95=paste0("[",ci.lower,",",ci.upper,"]"))%>%
select(Effect=rhs,b=est,SE=se,"z-value"=z,"p-value"=pvalue,"95%CI"=CI95)%>%
kable(caption="Indirecteffectregressionweightsand95%confidenceintervals",
digits=3)%>%
kable_styling(bootstrap_options="striped")%>%
write_file("cov_indirecteffects.html")
```

```
sem_vals<-lavaan::lavPredict(sem.cov.boot.seed, type="lv", method="regression") %>%
as.data.frame()


sem_vals <- sem_vals %>%
mutate(age = df$age,
       edu = df$edu_degree)
```

```
sem_vals %>%
cor() %>%
corrplot(order="hclust")
```

```
demog <-
df %>%
select(age, sex, country, risk, precond1, edu_degree)


head(demog)
```

```
# A tibble: 6 x 6
# Rowwise: 
    age   sex country  risk precond1 edu_degree
  <dbl> <dbl>   <dbl> <dbl>    <dbl>      <dbl>
1    28     0       0     0        0          0
2    19     0       0     0        0          0
3    36     0       1     0        0          1
4    52     1       0     0        0          1
5    35     0       1     0        0          1
6    20     1       1     0        0          0
```

## Describe Demographics of the Sample

```
demog %>% 
  select_if(is.numeric) %>% 
  psych::describe()
```

```
           vars   n  mean    sd median trimmed   mad min max range  skew
age           1 395 34.40 14.82     30   32.79 13.34  14  78    64  0.77
sex           2 395  0.68  0.47      1    0.72  0.00   0   1     1 -0.75
country       3 395  0.32  0.47      0    0.27  0.00   0   1     1  0.77
risk          4 395  0.13  0.34      0    0.04  0.00   0   1     1  2.20
precond1      5 395  0.12  0.33      0    0.03  0.00   0   1     1  2.31
edu_degree    6 395  0.44  0.50      0    0.43  0.00   0   1     1  0.24
           kurtosis   se
age           -0.61 0.75
sex           -1.44 0.02
country       -1.40 0.02
risk           2.86 0.02
precond1       3.34 0.02
edu_degree    -1.95 0.03
```

```
count(demog, sex)
```

```
# A tibble: 2 x 2
# Rowwise: 
    sex     n
  <dbl> <int>
1     0   128
2     1   267
```

```
count(demog, country)
```

```
# A tibble: 2 x 2
# Rowwise: 
  country     n
    <dbl> <int>
1       0   269
2       1   126
```

```
count(demog, edu_degree)
```

```
# A tibble: 2 x 2
# Rowwise: 
  edu_degree     n
       <dbl> <int>
1          0   221
2          1   174
```

```
count(demog, risk)
```

```
# A tibble: 2 x 2
# Rowwise: 
   risk     n
  <dbl> <int>
1     0   344
2     1    51
```

```
count(demog, precond1)
```

```
# A tibble: 2 x 2
# Rowwise: 
  precond1     n
     <dbl> <int>
1        0   347
2        1    48
```

```
sem_vals %>% 
  ggplot(aes(x = age, y = lonel)) +
    geom_jitter() +
    geom_smooth()
```

```
sem_vals <- sem_vals %>% 
  cbind(demog %>% select(-age)) %>% 
  select(-edu)
```

```
ggplot(sem_vals, aes(x = factor(edu_degree), y = lonel, fill = edu_degree)) +
    geom_violin(draw_quantiles = c(.5), alpha = .4) +
    geom_jitter(alpha = .25, width = .2) +
    theme(legend.position = "none")
```

```
pacman::p_load(apaTables)
```

```
package 'apaTables' successfully unpacked and MD5 sums checked

The downloaded binary packages are in
    C:\Users\b1003556\AppData\Local\Temp\Rtmp2bJiCQ\downloaded_packages
```

```
sem_vals %>% 
  select(discr, exp, BIS, BAS, react, lonel, own_proj, media, security, sysj, age, sex, country, risk, precond1, edu_degree) %>% 
  mutate(sex = recode(sex, "male" = 0, "female" = 1),
         country = recode(country, "DE" = 0, "AT" = 1)) %>% 
  apa.cor.table("cor_table.doc")
```

```
Means, standard deviations, and correlations with confidence intervals
 

  Variable       M     SD    1            2            3           
  1. discr       0.00  0.38                                        
                                                                   
  2. exp         -0.00 0.74  .09                                   
                             [-.00, .19]                           
                                                                   
  3. BIS         0.11  0.66  .89**        .11*                     
                             [.86, .91]   [.02, .21]               
                                                                   
  4. BAS         0.22  0.67  -.79**       -.15**       -.67**      
                             [-.82, -.75] [-.24, -.05] [-.72, -.61]
                                                                   
  5. react       -0.27 0.57  .88**        .11*         .83**       
                             [.86, .90]   [.01, .20]   [.80, .86]  
                                                                   
  6. lonel       -0.00 0.54  .39**        .05          .28**       
                             [.30, .47]   [-.04, .15]  [.19, .37]  
                                                                   
  7. own_proj    -0.37 0.45  -.28**       .10          -.18**      
                             [-.37, -.19] [-.00, .19]  [-.27, -.08]
                                                                   
  8. media       0.28  0.37  .24**        .12*         .42**       
                             [.15, .33]   [.03, .22]   [.33, .50]  
                                                                   
  9. security    0.26  0.34  .07          -.10*        .27**       
                             [-.03, .17]  [-.20, -.00] [.18, .36]  
                                                                   
  10. sysj       0.09  1.18  -.26**       .18**        -.22**      
                             [-.35, -.17] [.08, .27]   [-.31, -.13]
                                                                   
  11. age        34.40 14.82 -.04         -.02         -.08        
                             [-.14, .06]  [-.12, .08]  [-.18, .02] 
                                                                   
  12. sex        0.68  0.47  .06          .09          .15**       
                             [-.04, .16]  [-.01, .19]  [.05, .24]  
                                                                   
  13. country    0.32  0.47  -.08         .13*         -.07        
                             [-.18, .01]  [.03, .23]   [-.17, .03] 
                                                                   
  14. risk       0.13  0.34  .15**        -.13**       .11*        
                             [.05, .24]   [-.23, -.03] [.01, .21]  
                                                                   
  15. precond1   0.12  0.33  .06          -.08         .04         
                             [-.04, .16]  [-.17, .02]  [-.06, .14] 
                                                                   
  16. edu_degree 0.44  0.50  -.10         .03          -.05        
                             [-.19, .00]  [-.07, .13]  [-.15, .05] 
                                                                   
  4            5            6            7            8            9          
                                                                              
                                                                              
                                                                              
                                                                              
                                                                              
                                                                              
                                                                              
                                                                              
                                                                              
                                                                              
                                                                              
  -.69**                                                                      
  [-.74, -.64]                                                                
                                                                              
  -.43**       .36**                                                          
  [-.51, -.35] [.27, .44]                                                     
                                                                              
  .27**        -.07         -.27**                                            
  [.18, .36]   [-.17, .03]  [-.36, -.17]                                      
                                                                              
  -.04         .33**        -.08         -.07                                 
  [-.14, .06]  [.24, .42]   [-.18, .02]  [-.17, .03]                          
                                                                              
  .09          -.04         -.40**       .18**        .49**                   
  [-.01, .18]  [-.13, .06]  [-.48, -.31] [.08, .27]   [.41, .56]              
                                                                              
  .22**        -.29**       -.41**       .18**        .25**        .04        
  [.13, .32]   [-.38, -.20] [-.49, -.32] [.09, .28]   [.16, .34]   [-.06, .14]
                                                                              
  .17**        -.25**       -.16**       -.36**       -.25**       .09        
  [.07, .26]   [-.34, -.15] [-.26, -.07] [-.44, -.27] [-.34, -.15] [-.01, .19]
                                                                              
  -.10         .02          -.02         .06          .58**        .36**      
  [-.19, .00]  [-.08, .12]  [-.12, .07]  [-.04, .15]  [.51, .64]   [.27, .44] 
                                                                              
  .04          -.01         .10          .18**        .11*         -.08       
  [-.06, .14]  [-.11, .09]  [-.00, .19]  [.08, .28]   [.01, .20]   [-.18, .02]
                                                                              
  -.05         .01          .04          -.30**       -.04         .10*       
  [-.15, .04]  [-.09, .10]  [-.06, .14]  [-.39, -.21] [-.14, .06]  [.00, .20] 
                                                                              
  -.01         .00          -.03         -.14**       -.01         .08        
  [-.11, .09]  [-.10, .10]  [-.13, .07]  [-.24, -.04] [-.11, .09]  [-.02, .18]
                                                                              
  .16**        -.14**       -.10*        .01          -.03         .09        
  [.06, .25]   [-.23, -.04] [-.20, -.00] [-.09, .11]  [-.13, .07]  [-.00, .19]
                                                                              
  10           11           12           13           14          15         
                                                                             
                                                                             
                                                                             
                                                                             
                                                                             
                                                                             
                                                                             
                                                                             
                                                                             
                                                                             
                                                                             
                                                                             
                                                                             
                                                                             
                                                                             
                                                                             
                                                                             
                                                                             
                                                                             
                                                                             
                                                                             
                                                                             
                                                                             
                                                                             
                                                                             
                                                                             
                                                                             
                                                                             
                                                                             
  -.04                                                                       
  [-.14, .06]                                                                
                                                                             
  -.01         -.11*                                                         
  [-.11, .09]  [-.21, -.01]                                                  
                                                                             
  .07          -.27**       .10*                                             
  [-.03, .17]  [-.36, -.18] [.00, .20]                                       
                                                                             
  -.14**       .33**        .04          -.13**                              
  [-.24, -.04] [.24, .42]   [-.06, .14]  [-.23, -.04]                        
                                                                             
  -.07         .15**        -.04         -.07         .57**                  
  [-.17, .03]  [.05, .24]   [-.14, .06]  [-.17, .03]  [.50, .64]             
                                                                             
  .07          .25**        -.10*        -.06         -.10        -.00       
  [-.03, .17]  [.16, .34]   [-.20, -.01] [-.16, .04]  [-.20, .00] [-.10, .10]
                                                                             

Note. M and SD are used to represent mean and standard deviation, respectively.
Values in square brackets indicate the 95% confidence interval.
The confidence interval is a plausible range of population correlations 
that could have caused the sample correlation (Cumming, 2014).
* indicates p < .05. ** indicates p < .01.
```

## Calculate means to describe latent variables of the scales:

```
means <- tibble(.rows = nrow(df))

means$discrep <- df %>% 
  select(aut2, aut1, aut3, aut4, uncer1, uncer2, uncer3, uncer4, agenc1, agenc2, agenc3, agenc4) %>% 
  psych::alpha(check.keys = T) %>% 
  .$scores


means$exp <-
  df %>%
  select(starts_with("expec")) %>% 
    psych::alpha(keys = c(1, -1, -1), check.keys = F) %>% 
    .$scores


means$BIS <- df %>% 
  select(fear2, fear5, fear6, bis1, bis2, bis3, bis4, bis5) %>% 
  psych::alpha() %>% 
  .$scores

means$BAS <- 
  df %>% 
  select(starts_with("bash")) %>% 
    psych::alpha() %>% 
    .$scores

means$reac <- df %>% 
  select(reac2, reac1, reac3, reac4, reac5, reac6, reac7, host1, host2,host3, host4, host5, host6) %>% 
  psych::alpha(check.keys = T) %>% 
  .$scores


means$lonel <- df %>% 
  select(lsoc5, lsoc1, lsoc2, lsoc4, lsoc3, 
lfam1, lfam2, lfam3, lfam4, lfam5, lrom1, lrom2, lrom3, lrom4, lrom5) %>% 
  psych::alpha(check.keys = T) %>% 
  .$scores

means$own_pro <- df %>% 
  select(own_projects, doing_sports, creative) %>% 
  psych::alpha() %>% 
  .$scores

means$media <- df %>% 
  select(information, digital_social_contacts, social_media) %>% 
  psych::alpha() %>% 
  .$scores


means$security <- df %>% 
  select(washing_hands, disregard_curfew, stockpiling) %>% 
  psych::alpha(keys = c(1, -1, 1)) %>% 
  .$scores


means$sysj <- df$sysjust

means <- means %>% 
  cbind(., sem_vals %>% select(age, risk, precond1, edu_degree))
```

```
means %>% 
  describe()
```

```
           vars   n  mean    sd median trimmed   mad   min   max range  skew
discrep       1 395  2.93  0.58   2.92    2.92  0.62  1.08  4.83  3.75  0.09
exp           2 395  3.22  0.93   3.33    3.25  0.99  1.00  5.00  4.00 -0.30
BIS           3 395  2.22  0.81   2.00    2.13  0.74  1.00  4.88  3.88  0.86
BAS           4 395  2.98  0.78   3.00    3.00  0.89  1.00  4.80  3.80 -0.22
reac          5 395  1.87  0.53   1.77    1.81  0.46  1.00  4.38  3.38  1.36
lonel         6 395  5.56  1.01   5.73    5.64  1.09  1.53  7.00  5.47 -0.62
own_pro       7 395  3.74  0.73   3.78    3.78  0.82  1.56  5.00  3.44 -0.46
media         8 395  2.90  0.63   2.89    2.90  0.66  1.11  5.00  3.89 -0.02
security      9 395  3.40  0.47   3.44    3.43  0.33  1.78  4.67  2.89 -0.60
sysj         10 395  4.72  0.95   4.75    4.75  0.93  1.00  7.00  6.00 -0.41
age          11 395 34.40 14.82  30.00   32.79 13.34 14.00 78.00 64.00  0.77
risk         12 395  0.13  0.34   0.00    0.04  0.00  0.00  1.00  1.00  2.20
precond1     13 395  0.12  0.33   0.00    0.03  0.00  0.00  1.00  1.00  2.31
edu_degree   14 395  0.44  0.50   0.00    0.43  0.00  0.00  1.00  1.00  0.24
           kurtosis   se
discrep        0.11 0.03
exp           -0.47 0.05
BIS            0.32 0.04
BAS           -0.48 0.04
reac           2.71 0.03
lonel         -0.03 0.05
own_pro       -0.30 0.04
media         -0.15 0.03
security       0.44 0.02
sysj           0.24 0.05
age           -0.61 0.75
risk           2.86 0.02
precond1       3.34 0.02
edu_degree    -1.95 0.03
```

```
pacman::p_load(PerformanceAnalytics)
```

```
package 'xts' successfully unpacked and MD5 sums checked
package 'quadprog' successfully unpacked and MD5 sums checked
package 'zoo' successfully unpacked and MD5 sums checked
package 'PerformanceAnalytics' successfully unpacked and MD5 sums checked

The downloaded binary packages are in
    C:\Users\b1003556\AppData\Local\Temp\Rtmp2bJiCQ\downloaded_packages
```

```
png("corr_plot.png", res = 300, width = 3600, height = 3600)
chart.Correlation(means, histogram = T, )
dev.off()
```

```
png 
  2
```

```
save.image("workspace_s1.rdata")
```
