# Supplementary material for: From anxiety to action—Experience of threat, emotional states, reactance, and action preferences in the early days of COVID-19 self-isolation in Germany and Austria
Source: PLoS One. 2020 Dec 8;15(12):e0243193. doi: 10.1371/journal.pone.0243193 (PMC7723254; doi:10.1371/journal.pone.0243193)
Supplement: S2 File — (DOCX) [file pone.0243193.s002.docx]

# Supporting Information

## Survey Items and Material

### Discrepancy Scale

#### Motivational-affective discrepancy

Trotz des Coronavirus kann ich so ziemlich alles tun, was ich mir in den Kopf gesetzt habe.

Das Coronavirus bestimmt zu einem großen Teil, was ich tun kann und was nicht.

Wegen des Coronavirus liegt das, was in meinem Leben passiert, derzeit außerhalb meiner Kontrolle.

Das Coronavirus verhindert Dinge, die ich tun will.

Die Unvorhersehbarkeit des Coronavirus-Ausbruchs bringt mich nicht aus der Ruhe.

Während der Corona-Pandemie ist es frustrierend, nicht alle Informationen zu haben, die ich brauche.

Es nervt mich, dass mich der Ausbruch des Coronavirus überrascht hat.

Die Ungewissheit um das Coronavirus hält mich davon ab, ein erfülltes Leben zu führen.

Ich bezweifle, dass ich mit den unerwarteten Folgen des Coronavirus effizient umgehen kann.

Selbst wenn ich den notwendigen Aufwand investiere, kann ich die Probleme nicht lösen, die mit dem Coronavirus auftreten.

Ich kann während der Corona-Pandemie ruhig bleiben, weil ich mich auf meine Fähigkeiten verlassen kann, mit der Situation umzugehen.

Wenn mir das Coronavirus Probleme bereitet, bin ich sicher, dass ich eine Lösung dafür finde.

#### Epistemic Discrepancy

Der Verlauf der Corona-Pandemie hat mich überrascht.

Ich habe den weltweiten Ausbruch des Coronavirus erwartet.

Die aktuelle Coronasituation war vorhersehbar.

### Affect Items

#### Reactance

frei

eingeschränkt

frustriert

verärgert

illegitim behandelt

unangemessen behandelt

unter Druck

#### Fear

furchtsam

unsicher

verängstigt

angeekelt

#### Hostility

feindselig

hasserfüllt

reizbar

voller Verachtung

wütend

#### Sadness

alleine
einsam
niedergeschlagen

traurig

trübsinnig

#### BIS-Anxiety

gehemmt

ängstlich

nervös

unruhig

besorgt

#### BAS-Approach

voller Energie

kraftvoll

kompetent

zielorientiert

entschlossen

#### BAS-deactivated relaxation

entspannt

ruhig

friedlich

### Action ratings

#### Rating Questions:

Bitte geben Sie hier an, wie sinnvoll Sie die folgenden Handlungen momentan einschätzen

Bitte geben Sie hier an, wie sehr Sie in den nächsten Tagen folgenden Handlungen nachgehen wollen

Bitte geben Sie hier an, wie gut die folgenden Handlungen Ihnen helfen können, sich besser zu fühlen

#### Actions

##### Security-Related

Große Vorratskäufe tätigen

Regelmäßig mindestens 20 Sekunden lang die Hände waschen

Trotz Warnungen und Einschränkungen andere Menschen treffen

##### Personal Projects

Sport treiben

Eigenen Interessen und Projekten nachgehen

Kreativ sein

##### Social Media Use

Soziale Kontakte auf digitalen Wegen (wieder-)herstellen (Telefon, Skype, soziale Medien)

Ständig nach neuen Informationen suchen (online, TV, Radio)

Ständig soziale Medien aktualisieren

Wie gebannt vor dem Fernseher sitzen

##### Others

Intensiv Zeit mit den Menschen in meinem Haushalt verbringen (Quality Time)

Spirituelle oder religiöse Rituale

Versuchen, die Nachbarn zu unterhalten (mit Musik, Unterhaltungen am Balkon)

### System Justification

Generell finde ich unsere Gesellschaft fair.

In dieser Krise funktioniert das politische System so wie es sollte.

Unsere ganze Gesellschaft sollte radikal umstrukturiert werden.

Mein Land ist das beste Land, um hier zu wohnen.

Die meisten ergriffenen Maßnahmen dienen wirklich dem Allgemeinwohl.

Mit den Maßnahmen hat jeder eine faire Chance auf Gesundheit und Glück.

Unser gesellschaftliches System wird von Jahr zu Jahr schlechter.

Die Gesellschaft ist so aufgebaut, dass die Menschen in der Regel das bekommen, was sie verdienen.

### Loneliness

Ich fühle mich als Teil einer Gruppe von Freunden.

Meine Freunde verstehen meine Motive und die Art, wie ich denke.

Ich habe keine Freunde, die meine Ansichten teilen, aber ich wünschte, ich hätte sie.

Ich kann mich auf die Hilfe meiner Freunde verlassen

Ich habe keine Freunde, die mich verstehen, aber ich wünschte, ich hätte sie.

Ich fühle mich allein, wenn ich mit meiner Familie zusammen bin.

In meiner Familie gibt es niemanden, auf dessen Unterstützung und Ermutigung ich mich verlassen kann, aber ich wünschte, es gäbe jemanden.

Ich fühle mich meiner Familie nahe.

Ich fühle mich als Teil meiner Familie.

Meine Familie sorgt sich wirklich um mich.

Ich habe einen romantischen Partner, mit dem ich meine intimsten Gedanken und Gefühle teilen kann.

Ich habe einen romantischen oder ehelichen Partner, der mich so unterstützt und ermutigt, wie ich es brauche.

Ich wünschte, ich hätte eine befriedigendere romantische Beziehung.

Ich habe einen romantischen Partner, zu dessen Glück ich beitrage.

Ich habe ein unerfülltes Bedürfnis nach einer engen romantischen Beziehung.

### Knowledge Test

Es gibt bereits eine Impfung für

COVID-19, die noch im April 2020

verbreitet werden kann.

Gurgeln mit Salzwasser oder Ethanol

kann die Infektionsgefahr reduzieren.

Eine Maske kann zuverlässig vor COVID-

19-Infektionen schützen.

Der Hauptübertragungsweg von

COVID-19 ist Tröpfcheninfektion.

Der COVID-19-Virus ist eng mit dem

SARS-Virus verwandt.

In seltenen Fällen lässt sich das Virus

auch über Haustiere übertragen.

Antibiotika helfen nicht gegen COVID-19.

Nur Menschen mit schweren

Vorerkrankungen können daran sterben.

Man sollte Menschen mit einem

chinesischen oder ostasiatischen

Äußeren meiden.

Zur Reduktion von Ansteckungsgefahr

sollte man keine Briefe oder Pakete

empfangen.

### Infection Guess

Bitte schätzen Sie im folgenden Feld, wie viele von 100.000

Einwohnern in Ihrem Landkreis / politischem Bezirk aktuell mit

dem Coronavirus infiziert sind.
